# Maternal mortality estimation methodologies: a scoping review and evaluation of suitability for use in humanitarian settings

**DOI:** 10.1186/s13031-024-00636-y

**Published:** 2024-12-19

**Authors:** Blake Erhardt-Ohren, Sandra I. McCoy, Dennis M. Feehan, Rohini J. Haar, Ndola Prata

**Affiliations:** 1 Bixby Center for Population, Health and Sustainability, School of Public Health, University of California, Berkeley, Berkeley, USA; 2 Division of Epidemiology, School of Public Health, University of California, Berkeley, Berkeley, USA; 3 Department of Demography, University of California, Berkeley, Berkeley, USA

**Keywords:** Maternal mortality, Abortion, Termination of pregnancy, Refugee, Forcibly displaced, Measurement, Estimation, Multiple systems estimation, Capture recapture, RAMOS, Reproductive age mortality survey

## Abstract

**Background:**

Around the world, a maternal death occurs approximately every two minutes—most of these deaths are preventable. The maternal mortality ratio is a key indicator for the Sustainable Development Goals, yet we have no reliable way to estimate maternal deaths in refugee or internally displaced persons (IDP) camps and settlements. The goal of this study was to understand the methodologies most suited for adaptation for use to estimate the proportion of maternal mortality due to abortion complications in these settings.

**Methods:**

We conducted a scoping review of methodologies to estimate maternal mortality and evaluated them using a predetermined set of criteria. We evaluated nine original methodologies using eleven categories related to implementation in refugee or IDP camps and settlements*: data sources, definitions, sample size, timing of point estimate relative to data collection, bias, human resources, time needed for implementation, data collection training, statistical training, digitalization,* and *cost*. Each category could be assigned zero to four points, for a total score of 44 points. After evaluating each original methodology, we reviewed the original publication's citations or searched for other implementations through October 2022. We revised the original scores and developed a rank-order list of the methodologies according to their suitability for implementation in refugee and IDP camps.

**Results:**

We identified 124 publications that estimated maternal mortality. The Maternal Deaths from Informants/Maternal Death Follow on Review (MADE-IN/MADE-FOR) (33.5), hospital- or facility-based (33.5), and community informant-based (32.5) methodologies ranked highest due to low costs, short time interval needed for implementation, small sample sizes and close timing of point estimate relative to data collection, easy digitalization, and the need for no statistical training.

**Discussion:**

Similar to the lack of a “perfect” methodology to estimate maternal mortality in stable settings, there are compromises to consider when applying these methodologies to humanitarian settings. The most promising methodologies are adaptable to practical constraints in refugee and IDP camps and settlements. New methodologies that adapt and strengthen the MADE-IN/MADE-FOR, hospital- or facility-based, and community informant-based methodology show promise and must be further developed.

**Supplementary Information:**

The online version contains supplementary material available at 10.1186/s13031-024-00636-y.

## Plain language summary

Around the world, a maternal death occurs approximately every two minutes—most of these deaths are preventable. The goal of this study was to understand the methodologies most suited for use to estimate maternal deaths due to abortion complications in refugee and internally displaced persons (IDP) camps and settlements. We conducted a scoping review of methodologies to estimate maternal mortality and evaluated them using a predetermined set of criteria. We used eleven evaluation categories: *data sources, definitions, sample size, timing of point estimate relative to data collection, bias, human resources, time needed for implementation, data collection training, statistical training, digitalization*, and *cost*. Each category could be assigned zero to four points, for a total score of 44 points. We identified 124 publications that estimated maternal mortality. The Maternal Deaths from Informants/Maternal Death Follow on Review (MADE-IN/MADE-FOR) (33.5), hospital- or facility-based (33.5), and community informant-based (32.5) methodologies ranked highest due to low costs, short time interval needed for implementation, small sample sizes and close timing of point estimate relative to data collection, easy digitalization, and the need for no statistical training. Similar to the lack of a "perfect" methodology to estimate maternal mortality in stable settings, there are compromises to consider when applying these methodologies to humanitarian settings. The most promising methodologies are adaptable to practical constraints in refugee and IDP camps. New methodologies that adapt and strengthen the MADE-IN/MADE-FOR, hospital- or facility-based, and community informant-based methodology must be developed.

**Supplementary Information:**

The online version contains supplementary material available at 10.1186/s13031-024-00636-y.

## Background

In 2020, a maternal death occurred approximately every two minutes—for a maternal mortality ratio (MMR) of approximately 223 deaths per 100,000 live births globally [1]. These are deaths to women of reproductive age (aged 15–49 years old—WRA), who are pregnant, postpartum, post-abortion, or delivering, and whose death was caused by or aggravated by the pregnancy [2]. Approximately 75% of these deaths are caused by severe bleeding, infection, high blood pressure, other complications from delivery, and unsafe abortion [1]. Most of these deaths are preventable.

The 1999 annual UNFPA report states that “post-abortion complications account for some 25–50 per cent of maternal deaths in refugee situations” [3]. Globally, 7.9% of maternal deaths are attributable to unsafe abortion, which implies that the UNFPA estimate for abortion-related maternal mortality in refugee situations represents an increased risk of about three to six times [4]. Since the report's publication, this 25–50% estimate has been widely cited in peer-reviewed journal publications [5, 6], despite a lack of information in the report, including the methodology, geography, and time-period under review [3]. Although some studies have since attempted to estimate or understand maternal mortality in forcibly displaced populations [7–13], only one included abortion as a cause of death, though the investigators did not report any abortion deaths [8].

Decreased access to contraception and increased sexual violence put forcibly displaced individuals at a higher risk of unintended pregnancy. One in four of the over 89 million forcibly displaced individuals globally is at risk of unintended pregnancy [14]. Health facilities in humanitarian settings often experience stock outs of contraceptive methods or may not have the requisite healthcare providers or equipment to provide high-quality reproductive health services [15]. This reduced access to contraceptives may increase the number of unintended pregnancies in these populations [16]. Forcibly displaced individuals are more likely to experience sexual violence and exploitation than host communities or individuals in other settings [17]: in complex humanitarian emergencies, the reported prevalence of sexual-based violence is 21.4% [18], compared to about 10% globally [19].

Once forcibly displaced individuals become pregnant, they may face barriers to safe abortion care if they wish to end their pregnancy. A scoping review of barriers and facilitators to safe abortion care in humanitarian crises found that the legal environment, context, stigma, economic factors, and service delivery constraints prevent access to services [20]. While there is little research exploring forcibly displaced individuals' access to comprehensive abortion care, it is reasonable to expect that they may face issues similar or greater than the barriers that general populations face at the policy, community, health facility, interpersonal, and individual level [21–24]. The AMoCO study in Nigeria and the Central African Republic documented high levels of abortion-related morbidity and mortality in hospitals in fragile settings, demonstrating the challenges individuals in humanitarian crises face in accessing safe services [25].

Maternal mortality, and especially abortion complication-related mortality, is challenging to estimate even in stable populations [26–28]. While many methodologies have been created and tested in stable populations, they suffer from significant limitations. Direct methodologies measure mortality using vital registration systems or facility-based data and indirect methodologies rely on other techniques to elicit information about maternal deaths [28]. Methodologies that use routinely collected information may not find reliable data if there are large gaps in reporting or inaccurate reporting. Central to many of these challenges is that maternal death is relatively rare [2]. This means that many indirect methodologies require large samples to be considered reliable [28]. Methods that require surveying next of kin may suffer from response bias if there are no eligible respondents or selected households are unavailable for participation; when next of kin are available, social desirability bias may affect the cause of death reported. Even when social desirability bias is not an issue, respondents need to know about the decedent's death, their pregnancy status at the time of their death, and their cause of death, for enumerators to properly classify the death. Estimating abortion-related deaths is especially challenging given its taboo status in much of the world. Despite the constraints present in accurately estimating maternal mortality, the maternal mortality ratio is a key indicator for the Sustainable Development Goal #3.1, which calls for a reduction in global maternal mortality to less than 70 deaths per 100,000 live births by 2030 [29].

While past reviews have investigated methodologies to estimate maternal mortality in low- and middle-income countries [26–28] and humanitarian emergencies [30], the purpose of this paper is to (1) catalogue and review the existing methodologies for estimating maternal mortality, (2) understand their potential applicability to forcibly displaced populations, specifically refugee and internally displaced persons (IDP) in camps and settlements (hereafter referred to as “camps”), and (3) make a recommendation for future estimates of maternal mortality, and specifically maternal mortality due to abortion complications, in these populations. This information is imperative for monitoring and evaluating sexual and reproductive health programs in refugee and IDP settings.

## Methods

**Fig. 1:**
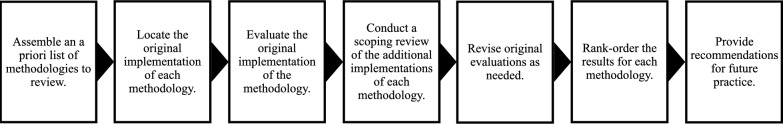
Process diagram of steps to review and evaluate methodologies to estimate maternal mortality

In brief, we conducted a scoping review of methodologies to estimate maternal mortality and evaluated them using a predetermined set of criteria. We created an a priori list of nine methodologies to estimate maternal mortality based on our experience in these settings and the literature on surveillance implementation in humanitarian settings [26–28]. Our strategy was to identify items for each of the methodologies currently in use. There are only a finite number of methodologies used to estimate maternal mortality, to date, so in essence we conducted a systematic search of each methodology, its adaptations, and subsequent implementation. We evaluated each of the original methodologies using eleven categories related to implementation in refugee or IDP camps*.* Each category could be assigned zero to four points, for a total score of 44 points. After evaluating each original methodology, we reviewed the original publication’s citations, and if there were not at least ten additional applications of the methodology, searched for other implementations through October 2022. We revised the original scores accordingly and developed a rank-order list of the methodologies according to their suitability for implementation in refugee and IDP camps. Figure 1 presents a process diagram of the different steps we took to evaluate the existing methodologies to estimate maternal mortality.

### Assemble a priori list of methodologies

We created an a priori list of methodologies based on our experience in the humanitarian sector that included: census- or survey-based, hospital- or facility-based methodology, Maternal Deaths from Informants/Maternal Death Follow on Review (MADE-IN/MADE-FOR), motherhood, neighborhood, prospective informant methodologies, community informant-based, Reproductive Age Mortality Survey (RAMOS), and direct sisterhood. The direct methodologies that we included in this review are motherhood, hospital- or facility-based, census- or survey-based, and direct sisterhood. The indirect methodologies included in this review are MADE-IN/MADE-FOR, neighborhood, indirect sisterhood, RAMOS, and community informant-based.

### Locate original implementation of the methodology

We located the original field implementation of each methodology by searching PubMed with the methodology name and reviewing article references to find the first instance when a methodology was cited; we continued the process until we found the article or another published item that explicitly stated it was presenting a novel methodology. If the original authors later issued corrections or changes to the methodology, we included these new publications as though they were part of the original methodology and revised our evaluation accordingly. Hospital- or facility- based, census- or survey-based, and community informant-based methodologies were considered not to have original implementations captured by specific research publications. We did not constrain original methodology searches by year or publication status, but excluded items that were not published in the English language and that were not instances of the methodology’s implementation.

### Evaluation of the original methodologies

Given the constraints that are present in refugee and IDP camps, we developed eleven criteria upon which to evaluate each methodology relevant to implementation in this specific environment for these unique populations, and as they relate to the specific task of identifying abortion-related deaths. We developed these criteria by reviewing the literature and through our own experience in humanitarian settings and estimating mortality due to abortion complications. Several of these criteria (1, 3, 4, 7, 8, 9, 10) deal explicitly with resource constraints that are common in humanitarian settings and the importance of training to assist enumerators with collecting data on sensitive topics, like abortion [31]. Others are concerned with the reliability of estimates (2, 5, 6, 9, 11) given refugee and IDP camps' open populations, the likelihood of incomplete data sets, and the added difficulty of estimating mortality due to abortion [32]. The criteria are listed below in Table 1*.*

**Table 1:** Criteria for evaluating methodologies to estimate maternal mortality in refugee and IDP camps in low resource settings

Criterion	Definition	Example	Relevance
Statistical training	The level of statistical knowledge required to implement the methodology and analyze results. Referent is an individual with basic arithmetic skills	Bayesian statistics, multilevel modeling	Camp settings: Statisticians with advanced knowledge and skills may not be readily available
Bias	The extent to which the methodology introduces bias that may affect internal or external validity	Internal: misclassification bias that results in some deaths being counted or not counted as maternal deaths and/or abortion-related; selection bias that prevents certain individuals’ deaths from being counted External: generalizability that prevents results from being widely interpreted	Abortion: Informants reporting on a maternal death must know about a pregnancy and circumstances of a death to be able to report accurately if it is abortion-related. Due to abortion stigma, deaths that are known to be due to abortion may not be reported as such
Cost	The total cost to implement the methodology, including human resources' level of effort and data collection and analysis tools. This cost excludes transportation and incidentals for human resources	X% of total maternal health costs, x total US$	Camp settings: There may not be sufficient budgets in humanitarian response for expensive surveillance systems for monitoring and evaluation
Data collection training	The amount of training required for an individual with no background in data collection	Survey methodology, interview techniques, disease identification	Abortion: Due to stigmatization of abortion, enumerators must be sensitized to the topic and trained on how to collect data without introducing bias
Data sources	Whether a single or multiple data sources are required. The type of data source used for the estimation of maternal mortality	Facility records, death records, verbal autopsy	Camp settings: Data sources may be incomplete or missing Abortion: Due to stigmatization of abortion, deaths due to abortion complications in facilities may be coded incorrectly. Reports about deaths from informants may be inaccurate for the same reason
Definitions	Whether the data source includes a definition of maternal mortality that is consistent with the World Health Organization (WHO) definition [2] or a different maternal death definition	Deaths of pregnant individuals according to International Classification of Diseases (ICD) 10 [33] or ICD 11, [34] any death of a reproductive age individual, etc	Abortion: To accurately capture abortion-related deaths, definitions must specify abortion as a cause of death
Digitalization	Whether the methodology can be digitized into an existing open-source platform for data collection, management, and analysis	Google forms, [35] DHIS2 program, [36] Open Data Kit [37] or KoboToolbox [38] questionnaire	Camp settings: Digitalization of methodologies may simplify data collection during chaotic humanitarian responses Abortion: Using digital data collection methods, may improve ability to collect information confidentially through informants' direct interaction with questionnaires or other data collection forms, protecting them from stigma associated with abortion
Human resources	The number of individuals and their time required to implement the methodology relative to sample size	X clinicians, x health facility managers, x community health workers, etc. for x days	Camp settings: Due to competing priorities, human resources may not be readily available
Sample size	The minimum number of maternal deaths and/or abortion-related deaths required by the methodology to produce a precise estimate	X total maternal deaths, x maternal deaths attributable to abortion complications	Camp settings: These settings may not have large sample sizes needed to generate estimates using certain methodologies Abortion: Since the contribution of abortion-related complications to the structure of causes of maternal mortality in humanitarian settings is unknown, methodologies may need to be able to utilize small numbers to produce estimates
Time needed for implementation	The amount of time required to implement the methodology and produce results	X days, weeks, months, years	Camp settings: In rapid responses, it may be important to implement methodologies quickly, in order to establish a baseline for monitoring and evaluation of sexual and reproductive health service provision
Timing of point estimate relative to data collection	The amount of time between the end of the time period used for analysis and the estimate of maternal mortality	Methodology produces an estimate of mortality in last x months/weeks/days after x months/weeks/days of methodology implementation	Camp settings: In rapid responses, it may be important to implement methodologies quickly, in order to establish a baseline for monitoring and evaluation of sexual and reproductive health service provision

We evaluated the methodologies by assigning between zero and four points—zero to indicate the lowest possible score and four to indicate the highest possible score—with regard to their suitability to implementation in refugee and IDP camps to estimate proportion of maternal deaths due to abortion complications in relation to other methodologies. The total possible number of points, or highest score, was 44, and categories were not weighted. For the first two methodologies under review, NP and BEO independently assigned scores, compared results, and recalibrated ratings. For all other methodologies, BEO evaluated the methodologies, NP confirmed scoring, and DF, RH, and SM reviewed scores for inconsistencies.

### Scoping review of additional implementations

To locate additional implementations of a given methodology, we first reviewed the citations for each original implementation listed in PubMed, Google Scholar, and Research Gate. If the original implementation did not have at least ten citations, we conducted additional searches on the same databases for the time period through October 2022, and evaluated as many implementations as were available. We selected a minimum of ten citations before conducting additional searches to try to capture a broad application of the methodology. The terms for the methodologies where we conducted additional searches are available in Table 2*.* Since hospital- or facility-based, census- or survey-based, and community informant-based methodologies were not considered to have original implementations, all data for these methodologies were collected through this search methodology. We did not constrain results by type of publication. Any publication that used the methodology in question was retained. We also included items located through references reviews of items located in each search.

**Table 2:** Search terms used on PubMed to locate additional implementations of each methodology

Methodology to estimate maternal mortality	Search terms
Census- or survey-based	(“census-based” or “survey-based”) and (“maternal death” or “maternal mortality”)
Community informant-based	((“informant” or “CHW” or “health worker”) and (“surveillance” or “prospective”)) and (“maternal death” or “maternal mortality”)
Hospital- or facility-based	(“hospital-based” or “facility-based”) and (“maternal death” or “maternal mortality”)
MADE-IN/MADE-FOR	“MADE-IN/MADE-FOR” and (“maternal death” or “maternal mortality”)
Motherhood	“motherhood method” and (“maternal death” or “maternal mortality”)
Neighborhood	(“neighborhood” or “community knowledge”) and (“maternal death” or “maternal mortality”)

### Evaluation of additional implementations

For this step in the assessment process, we evaluated each new implementation of a given approach according to the eleven categories and considered whether specific adaptations in each implementation, if any, strengthened or weakened the original methodology vis-à-vis its suitability to estimate the number of maternal deaths and proportion of maternal deaths due to abortion complications in refugee and IDP camps. If subsequent implementations strengthened the original methodology, we adjusted the score up for the relevant evaluation category; if they weakened the original methodology, we did not adjust the original score.

### Rank-order list creation

After completing the evaluation of original methodologies and subsequent implementations, we consolidated the results for each methodology, incorporating strengthening elements from implementations into the original methodology. We weighed each evaluation category evenly in the final assessment. We calculated the total number of points per methodology by summing the value assigned to each category and ranked the methodologies accordingly.

### Reporting

We report the results of the scoping review according to the Preferred Reporting Items for Systematic reviews and Meta-Analyses extension for Scoping Reviews (PRISMA-ScR) Checklist [39]. The methodologies are grouped into three sets in the Results: those with the highest scores, those with average scores, and those with the lowest scores.

## Results

We reviewed and evaluated nine methodologies to estimate maternal mortality: motherhood, hospital- or facility-based, census- or survey-based, direct sisterhood, MADE-IN/MADE-FOR, neighborhood, indirect sisterhood, RAMOS, and community informant-based. Of the methodologies with original implementations, all but one were implemented first in Africa and/or Asia—the first implementation of the direct sisterhood method also included data from Bolivia in addition to Sudan and Egypt. Table 3 lists the original methodologies, whether the methodology produces a direct or indirect estimate, the publication title, year of publication, authors, geography of the original implementation, and final score. Table 4 presents the mean, median, and range of scores for each criterion.

**Table 3:** Original methodologies evaluated in this scoping review, listed by total score, from highest to lowest ranking

Methodology name	Type of estimate	Summary of methodology	Original publication(s)	Year(s)	Authors	Geography	Score
The Maternal Deaths from Informants/Maternal Death Follow on Review (MADE-IN/MADE-FOR)	Indirect	MADE-IN: health volunteers and community level volunteers conduct listing-meetings with village informants to gather information on WRA deaths. Lists are consolidated. MADE-FOR: verbal autopsy is used with a relative of the decedent to specify cause of death	An option for measuring maternal mortality in developing countries: a survey using community informants [40]	2010	Qomariyah, SN, Baunholtz, D, Achiadi, EL, et al	Indonesia	33.5
Hospital- or facility-based	Direct	Researchers review deaths at healthcare facilities, sometimes with the use of a maternal death review committee	NA	NA	NA	NA	33.5
Community informant-based	Indirect	Individuals embedded within a community identify births and deaths in a community retrospectively or prospectively	NA	NA	NA	NA	32.5
Motherhood	Direct	Pregnant women meet regularly. Volunteers collect infant vaccination records from registries and information about deaths from group members. If there is no consensus about cause of death, verbal autopsies with household members	Field test results of the motherhood method to measure maternal mortality [41]	2011	Maskey, MK, Baral KP, Shah R, et al	Nepal	29.5
Neighborhood	Indirect	Adult women in a public setting are asked about deaths and births within a small, specified area, i.e., neighborhood, around their home	The neighbourhood method for measuring differences in maternal mortality, infant mortality and other rare demographic events [42]	2014	Alam, N, & Townend, J	Bangladesh	29
Indirect sisterhood	Indirect	Random sample of WRA are interviewed. Enumerators ask four questions to illicit deaths to ever-married female siblings of respondents	Estimating maternal mortality: the sisterhood method [43]	1989	Graham, W, Brass, W, & Snow, RW	The Gambia	26
Reproductive Age Mortality Study (RAMOS)	Indirect	Vital registration records identify deaths to WRA. Interviewers survey household members to determine cause of death	Reproductive mortality in two developing countries [44] Causes of death to women of reproductive age in two developing countries [45] Maternal mortality in Indonesia and Egypt [46] A comparison of two cause-of-death classification systems for deaths among women of reproductive age in Menoufia, Egypt [47]	1986 1987 1988 1988	Fortney JA, Susanti I, Gadalla S, et al Fortney JA, Gadalla S, Saleh S, et al Fortney JA, Susanti I, Gadalla S, et al Grubb GS, Fortney JA, Saleh S, et al	Egypt & Indonesia Egypt & Indonesia Egypt & Indonesia Egypt	23.5
Census- or survey-based	Direct	Nationally representative surveys or censuses that rely on vital statistics and civil registration data to develop sampling frames	NA	NA	NA	NA	22.5
Direct sisterhood	Direct	Household questionnaire collects a list of all brothers and sisters of respondents and their survival status	Direct and indirect estimates of maternal mortality from the sisterhood method	1991	Rutenberg, N, Sullivan, JM, & Demographic and Health Surveys	Bolivia, Sudan, & Egypt	22.5

**Table 4:** Mean, median, and score range for each criterion included in our evaluation

Criterion	Mean	Median	Range
Statistical training	2.0	2	(1, 3)
Bias	2.5	3	(1, 4)
Cost	2.2	3	(1, 4)
Data collection training	1.7	2	(1, 4)
Data sources	1.9	2	(1, 2.5)
Definitions	1.8	2	(1, 3.5)
Digitalization	2.0	2.5	(1, 3)
Human resources	2.7	3	(2, 4)
Sample size	3.6	3.5	(3, 4)
Time needed for implementation	4	4	(4, 4)
Timing of point estimate relative to data collection	2.2	3	(1, 4)

**Fig. 2:**
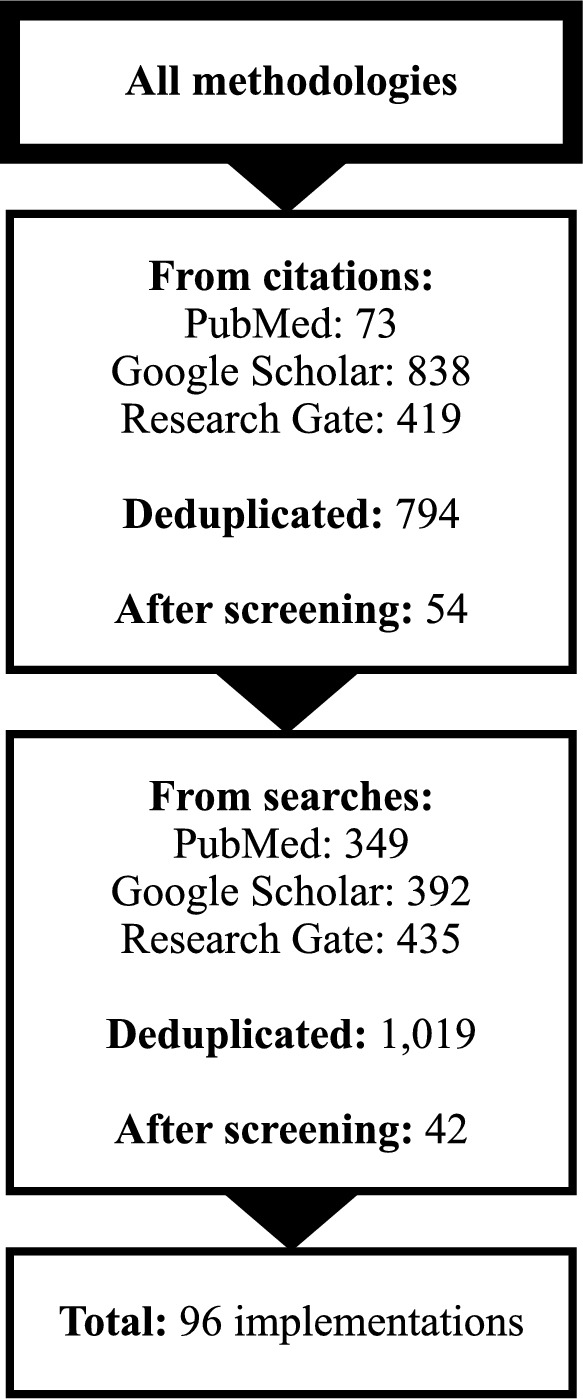
Flow diagram with summarized sources of additional implementations of all methodologies under review

**Fig. 3:**
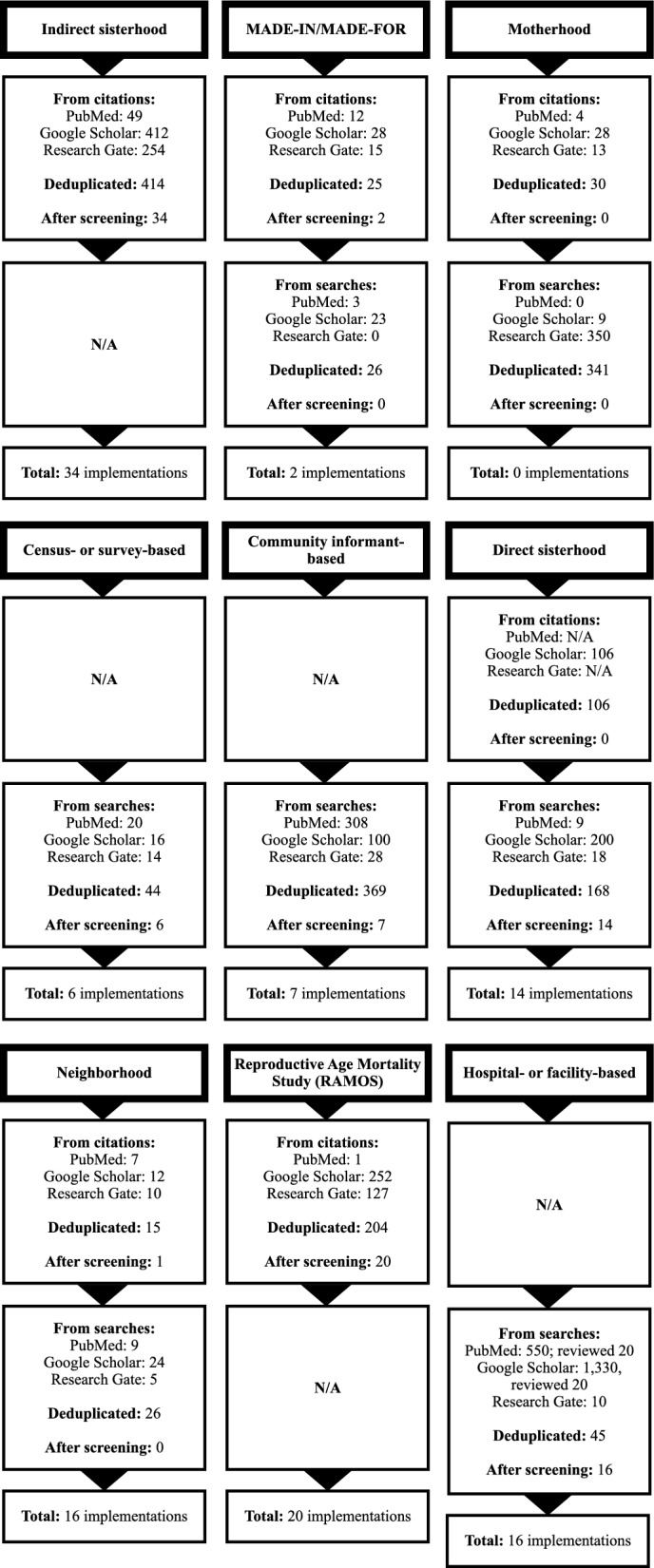
Flow diagram with sources of additional implementations of each methodology under review

We conducted additional searches for the MADE-IN/MADE-FOR, motherhood, and neighborhood methodologies. For MADE-IN/MADE-FOR, we found two items through citation reviews and no additional items from searches. For the motherhood methodology, we did not find any additional implementations from citations or searches. For the neighborhood methodology, we found one relevant citation and no additional items through searches. For the methodologies considered not to have original implementations (census- or survey-based, community informant-based, and hospital- or facility-based), we reviewed six, seven, and sixteen items from searches, respectively. We retrieved 34 articles and twenty additional articles from citation reviews for the indirect sisterhood and RAMOS methodologies. We identified no items from citations and fourteen items from searches for the direct sisterhood methodology. Figure 2 shows a summarized flow diagram for all methodologies and Fig. 3 for each methodology individually. A complete list of the publications considered as implementations of each original methodology is available in Additional file 1*.*

### Highest scores

The methodologies that received the highest scores after review of both the original implementation and subsequent implementations were MADE-IN/MADE-FOR (33.5), hospital- or facility-based (33.5), and community informant-based (32.5). The evaluation forms, with scores, for each of these three methodologies are available in Additional files 2–4. These methodologies all scored highly for *sample size* (4, 4, 4 points respectively for MADE-IN/MADE-FOR, hospital- or facility-based, and community informant-based), *timing of point estimate relative to data collection* (3, 3, 4), *time needed for implementation* (2.5, 3, 3), *statistical training* (3, 4, 4), *digitalization* (4, 4, 4), and *cost* (3, 4, 3). These methodologies have small *sample size*s and can be quickly *digitized* using data collection forms which require no *statistical training* to implement. Due to the way these data are collected, the *cost* to implement the methodologies is relatively low compared to other methodologies, and because estimates can be generated after one or two meetings (MADE-IN/MADE-FOR), retrospective review (hospital- or facility-based), or after retrospective data collection or ongoing prospective data collection (community informant-based), the *time needed for implementation* is insubstantial and the *timing of point estimate relative to data collections* is very close to the time of data collection.

The highest scoring methodologies differed in several other evaluation categories. While MADE-IN/MADE-FOR scored 3 points for *data sources*; hospital- or facility-based and community informant-based methodologies scored 1 and 2 points, respectively. This is because MADE-IN/MADE-FOR requires at least two data sources (reports from healthcare workers and verification through verbal autopsy), whereas the hospital- or facility-based methodology requires only facility records, though maternal death review committees can be involved, and the community informant-based methodology can use a myriad of sources to identify deaths within a population. *Definitions* scores likewise differed, with the methodologies receiving 3, 2, and 2 points, respectively. While MADE-IN/MADE-FOR provides a clear and standardized definition of pregnancy-related or maternal deaths, hospital- or facility-based and community informant-based methodologies provided definitions that were not standard and/or not followed in the methodologies' actual implementation. In terms of *bias*, the hospital- or facility-based methodology received one point because deaths may not be reported to a facility and then would not be included in counts, causing selection bias, whereas community informant-based and MADE-IN/MADE-FOR methodologies received 2.5 points due to their use of multiple sources with ongoing reporting to minimize recall bias; while these two latter methodologies include additional data sources for cause of death, they assume that recall among next of kin is accurate. While the hospital- or facility-based methodology received four points for *data collection training*, due to the straightforward nature of collecting data from maternal death records, MADE-IN/MADE-FOR received 3.5 points, and the community informant-based methodology received 2 points. Similarly, the hospital- or facility-based methodology received 3.5 points for human resources, due to the small amount of data collection, management, and analysis required, whereas the community-informant based methodology and MADE-IN/MADE-FOR received two points apiece due to more intensive staff needs.

### Average scores

The methodologies that received the average scores after review of both the original implementation and subsequent implementations were motherhood (29.5), neighborhood (29), and indirect sisterhood (26). The evaluation forms for each of these three methodologies, with scores, are available in Additional files 5–7. These methodologies all scored highly for *data collection training* (4, 3, 3 points respectively for motherhood, neighborhood, and indirect sisterhood), *statistical training* (4, 4, 3), *digitalization* (4, 4, 4), and *sample size* (3, 3, 2). While the original neighborhood methodology cites a half-day training requirement [42] and the original indirect sisterhood methodology makes mention of a one-hour training plus a half-day of supervision [48], the motherhood methodology does not require training [41]—thus, *data collection training* for these methodologies are simple to implement. Likewise, the calculation for maternal deaths in each of these three methodologies is clear and straightforward, requiring no *additional statistical training*. Due to the ease with which data are programmed into pre-existing digital tools, these methodologies are easily *digitized* for data collection. Neighborhood and motherhood methodologies received three points for *sample size* due to the lack of a prerequisite number of deaths for the methodologies to be implemented, whereas indirect sisterhood has a larger sample size requirement of 3000–6000 in most instances, but documentation provides clear guidance on calculation.

The motherhood, neighborhood, and indirect sisterhood methodologies similarly scored two points or fewer for *data sources* (2, 2, 2 points respectively)*, timing of point estimate relative to data collection* (1, 1, 1)*,* and *bias* (2, 2, 2)*.* Each of the three methodologies utilizes at least two *data sources*—for neighborhood, information is elicited through discussions with community members and confirmed via verbal autopsies, within the motherhood methodology, pregnant individuals share information that is verified with vaccination records and verbal autopsies with next of kin, and in indirect motherhood, census records lead to representative surveys of households in a given geography. All three methodologies received one point for the *timing of point estimate relative to data collection* due to the significant difference between implementation and when the estimate is valid: for the motherhood methodology, 2 years and 3 months before the study period, for the neighborhood method, 2 years and 42 days, and for indirect sisterhood, 5 years plus the amount of time spent in implementation. Lastly, each methodology received two points for *bias* due to the risk of selection bias, non-response bias, and recall bias.

The motherhood, neighborhood, and indirect sisterhood methodologies were similar in their middling scores in the *human resources* (3, 2.5, and 2 points respectively)*, time needed for implementation* (2.5, 2.5, 2)*, and cost* categories (3, 3, 2)*.* For *human resources*, the fewest number of individuals are needed for the motherhood methodology, where facilitators lead group discussions and occasionally collect verbal autopsies, as opposed to the *neighborhood method*, where enumerators gather information on deaths in public places and then routinely collect verbal autopsies, and the indirect sisterhood methodology, wherein households are randomly sampled for participation in the survey without knowing if/when maternal or other deaths will be reported. In terms of *time needed for implementation*, both the neighborhood and motherhood methodologies report the amount of time for the original studies, 42 days and 30 days, respectively, and then estimates for other studies, whereas the indirect sisterhood method suggests that for a shorter implementation time, a large cadre of staff are needed. While cost is not reported, it can be extrapolated from other information that indirect sisterhood would be the most expensive of these three methodologies, though neighborhood and motherhood methodologies also require considerable inputs for data collection and processing.

The methodologies differed most in scoring for *definitions* (1, 3, 3 points respectively for neighborhood, motherhood, and indirect sisterhood)*.* While the indirect sisterhood, at least in later implementations, and neighborhood methodologies provide clear definitions of maternal death in line with the International Classification of Diseases (ICD)-10 and standard WHO maternal death classifications, the motherhood methodological paper does not describe the deaths that they are looking, other than to say that they are maternal—it is unclear whether participants know how to identify these deaths too.

### Lowest scores

The methodologies that received the lowest scores after review of both the original implementation and subsequent implementations were RAMOS (23.5), census- or survey-based (22.5), and direct sisterhood (22.5). The evaluation forms for each of these three methodologies, with scores, are available in Additional files 8–10.

There are only two categories in which all three of these methodologies scored highly: *definitions* (4, 3, 3 respectively for RAMOS, direct sisterhood, and census- or survey-based) and *digitalization* (4, 4, 4). All surveys received high scores for *definitions* due to the consistent use of WHO standard maternal death definitions in line with ICD-10; each specific implementation of RAMOS methodology stated the exact deaths they were targeting. For *digitalization*, the methodologies all received four points because the tools are already digitized through the Demographic and Health Surveys and online verbal autopsy tools, which can be easily adapted by other implementers.

The methodologies all received two or fewer points for *sample size* (1, 1, 1 points respectively for RAMOS, direct sisterhood, and census- or survey-based methodologies)*, human resources* (1, 1, 1)*, time needed for implementation* (2, 1, 1)*, cost* (2, 1, 1), *data collection training* (2, 2, 2)*, timing of point estimate relative to data collection* (1, 2, 2)*,* and *bias* (2, 2, 2). Each of these methodologies require large *sample sizes* with multiple thousands of respondents to surveys, which is why each received only one point in this category. These large sample sizes require extensive *human resources,* including field supervisors and teams of enumerators. With large sample sizes and despite sizeable study teams, the *time needed for implementation* can be prolonged, especially in wide-ranging geographies. Though for the Demographic and Health Surveys (DHS), large sample sizes are related to the nationally-representative nature of the surveys, the direct sisterhood methodology still requires large numbers of respondents for precise estimates. These factors all lead to increased *cost* for these methodologies compared to other methodologies for estimating maternal mortality. Due to the vast time range (5 + years) for which respondents are requested to provide information about maternal deaths, each methodology received low scores for *timing of point estimate relative to data collection*, with RAMOS receiving two points due to the flexibility of the methodology to adjust for shorter time periods. Similar to all other methodologies, RAMOS, direct sisterhood, and census- or survey-based methodologies may be affected by significant levels of recall, non-response, and selection bias unless they are well-designed even in ideal scenarios.

In terms of *data sources*, the RAMOS methodology received three points, whereas direct sisterhood and census- or survey-based methodologies received two points. The RAMOS methodology calls for the triangulation of data from several sources, while census- or survey-based and direct sisterhood methodologies rely only on accurate census data and interviews with members of randomly selected households from a sampling frame. Due to the extremely simple and direct calculation of maternal mortality from census- or survey-based and direct sisterhood methodologies, they each received 3.5 points for *statistical training*, whereas the RAMOS methodology received only one point due to the lack of explanation for how the calculation is derived from the collected data in the original or any subsequent implementations of the methodology.

## Discussion

In this scoping review, we synthesize nine methodologies to estimate maternal mortality and abortion-related maternal deaths and determine their suitability for use in refugee and IDP camps in resource-scare settings. We do this by using eleven categories that account for practical implementation constraints in these environments and specifically for estimates of abortion-related mortality. The results of the review point to the tradeoffs inherent in existing methodologies—tensions between methodologies that are simple to implement and less accurate and those that are more technically complex but produce more reliable estimates. Even in stable settings, choosing the right methodology to estimate maternal mortality can be difficult, and these difficulties are amplified in humanitarian emergencies, including forcibly displaced persons camps. Resource scarcity, competing priorities, and incomplete data sets can make data collection especially challenging. Stigma associated with abortion may influence enumerator and provider recorded and informant given information about cause of death. While other reviews have evaluated these methodologies more broadly [26–28, 49], and despite the challenges of calculating maternal mortality estimates in these settings, we completed this review because believe that these figures are necessary to accurately understand and respond to the needs of refugee women and girls.

These tradeoffs are readily apparent in the top scoring methodologies in this scoping review. The MADE-IN/MADE-FOR methodology relies on more *data sources* than hospital- or facility-based methodology or the community informant-based methodology, which causes MADE-IN/MADE-FOR *costs* to be higher and require more *human resources*. On the other hand, MADE-IN/MADE-FOR and the community informant-based methodology are less likely to introduce bias to estimates than the hospital- or facility-based methodology. In these methodologies there are clear differences in the comprehensiveness of the approach and the time intensity to accomplish a more thorough estimate. Humanitarian practitioners will need to take these limitations into account when designing studies to estimate maternal mortality.

Though there are risks and benefits to the use of any of these methodologies in any setting, there is an opportunity to test new methodologies that combine the best qualities of the existing ones. For example, novel methodologies could investigate what *biases* may be introduced when using *data sources* that tap into the expertise of community members, such as community leaders, health workers, or pregnant mothers, and more readily available data, such as death records available in health facilities. While this may introduce new biases, it may also prevent *bias* resulting from incomplete data sets in humanitarian emergency settings by capturing deaths that may or may not be reported to authorities. Methodologies with clear *definitions* of maternal death and cause of death that use straightforward *data collection training* that are easily *digitized* due to their lack of *advanced statistics* may be more practical in non-stable settings where there are competing priorities.

Any adaptations or new methodologies should be tested and validated against stable-setting “gold standard” methodologies, i.e., RAMOS or census- or survey-based methodologies, in refugee and IDP camps. We also believe that any study using human subjects should undertake the most caution to ensure confidentiality and data privacy, especially when dealing with sensitive topics like maternal death and deaths due to abortion complications. Though we certainly can’t solve all issues related to the trickiness of estimating maternal mortality in these special circumstances, there are adaptations and opportunities worth exploring to estimate this significant indicator in refugee and IDP camps.

### Limitations

This study has several limitations. We conducted a scoping review of the literature, and not a systematic review, so we may have missed some methodologies. Similarly, we limited our review to published literature and items that were published in English, which means that grey literature and items in other languages were excluded. In almost all cases, we extrapolated cost based on other information provided in manuscripts, because many studies did not directly report expenses in publications—however, the results remain the same whether or not cost is included in the final score. Another limitation is that although this is an evaluation and ranking for refugee and IDP camps, we did not actually test each methodology in these environments. Instead, we reviewed the available evidence for each methodology, and using our expertise in humanitarian emergencies, considered and assessed using pre-determined categories, the application of each methodology in these populations. Ideally, we would implement all of these methodologies in a site with known mortality and determine their accuracy compared to the truth. This scoping review is subjective—we, the authors, determined the categories as well as their relative ranking, based on our expertise in humanitarian settings. Despite these limitations, the scoping review reveals insights into potential feasibility and applicability of existing methodologies to measure maternal mortality in refugee and IDP settings.

## Conclusions

To adequately address the specific sexual and reproductive health needs of pregnant and postpartum forcibly displaced persons, we must be able to accurately estimate the burden of maternal mortality in the population, and specifically, maternal mortality due to abortion complications. Three methodologies currently used to estimate maternal mortality and mortality related to abortion complications show the most promise for suitability to use in refugee and IDP settings. Thus, it is imperative to develop new methodologies that adapt and strengthen the MADE-IN/MADE-FOR, hospital- or facility-based, and community informant-based methodologies for research in forcibly displaced settings.

## Supplementary Information

Additional file 1. Publications included as additional implementations of each methodology under review. Additional file 1 is a complete list of the publications considered as implementations of each original methodology.

Additional file 2. MADE-IN/MADE-FOR methodology completed evaluation form. Additional file 2 shows the completed evaluation form for the MADE-IN/MADE-FOR methodology.

Additional file 3. Hospital- or facility-based methodology completed evaluation form. Additional file 3 shows the completed evaluation form for the hospital- or facility-based methodology.

Additional file 4. Community informant-based methodology completed evaluation form. Additional file 4 shows the completed evaluation form for the community informant-based methodology.

Additional file 5. Neighborhood methodology completed evaluation form. Additional file 5 shows the completed evaluation form for the neighborhood methodology.

Additional file 6. Motherhood methodology completed evaluation form. Additional file 6 shows the completed evaluation form for the motherhood methodology.

Additional file 7. Indirect sisterhood methodology completed evaluation form. Additional file 7 shows the completed evaluation form for the indirect sisterhood methodology.

Additional file 8. Reproductive Age Mortality Study (RAMOS) methodology completed evaluation form. Additional file 8 shows the completed evaluation form for the Reproductive Age Mortality Study (RAMOS) methodology.

Additional file 9. Census- or survey-based methodology completed evaluation form. Additional file 9 shows the completed evaluation form for the census- or survey-based methodology.

Additional file 10. Direct sisterhood methodology completed evaluation form. Additional file 10 shows the completed evaluation form for the direct sisterhood methodology.

## Data Availability

All data generated or analyzed during this study are included in this published article and its additional files.

## Abbreviations

DHS: Demographic and health surveys
ICD: International classification of diseases
IDP: Internally displaced person(s)
MADE-IN/MADE-FOR: Maternal deaths from informants/maternal death follow on review
NA: Not any
MMR: Maternal mortality ratio
PRISMA-ScR: Preferred reporting items for systematic reviews and meta-analyses extension for scoping reviews
RAMOS: Reproductive age mortality survey
WHO: World Health Organization
WRA: Woman/women of reproductive age
