# Supplementary material for: Maternal mortality estimation methodologies: a scoping review and evaluation of suitability for use in humanitarian settings
Source: Confl Health. 2024 Dec 19;18:75. doi: 10.1186/s13031-024-00636-y (PMC11657123; doi:10.1186/s13031-024-00636-y)
Supplement: Supplementary file 1 — Additional file 1. Publications included as additional implementations of each methodology under review. Additional file 1 is a complete list of the publications considered as implementations of each original methodology. [file 13031_2024_636_MOESM1_ESM.docx]

**Additional file 1. Publications included as additional implementations of each methodology under review**

| **Methodology name** | **Title(s) of publication(s)** | **Year(s) of publication** | **Author(s)** | **Geography** |
| --- | --- | --- | --- | --- |
| Census- or survey-based | *Every death counts: Measurement of maternal mortality via a census*  *How should we measure maternal mortality in the developing world? A comparison of household deaths and sibling history approaches.*  *Estimating pregnancy-related mortality from census data: Experience in Latin America*  *Measuring maternal mortality through the census: Rapier or bludgeon?*  *Undertaking cause-specific mortality measurement in an unregistered population: An example from Tigray Region, Ethiopia.*  *Rural-urban differentials in pregnancy-related mortality in Zambia: Estimates using data collected in a census* | 2001  2006  2009  2011  2014  2015 | Stanton C, Hobcraft J, Hill K, et al.  Hill K, El Arifeen S, Koenig M, Al-Sabir A, Jamil K, & Raggers H.  Hill K, Queiroz BL, Wong L, et al.  Hill K & Stanton C.  Godefay H, Abrha A, Kinsman J, Myléus A, & Byass P.  Banda R, Fylkesnes K, & Sandøy IF. | Benin, Iran, Laos, Madagascar, Zimbabwe  Bangladesh  Honduras, Nicaragua, Paraguay  Laos, Lesotho, Zimbabwe  Ethiopia  Zambia |
| Community informant-based | *Improving surveillance for maternal and perinatal health in 2 districts of rural Tanzania*^1^  *A prospective key informant surveillance system to measure maternal mortality - findings from indigenous populations in Jharkhand and Orissa, India*^2^  *An innovative approach to measuring maternal mortality at the community level in low-resource settings using mid-level providers: a feasibility study in Tigray, Ethiopia*^3^  *Implementation and utilisation of community-based mortality surveillance: A case study from Chad*^4^  *Community-linked maternal death review (CLMDR) to measure and prevent maternal mortality: a pilot study in rural Malawi*^5^  *Maternal mortality in rural south Ethiopia: outcomes of community-based birth registration by health extension workers*^6^  *A prospective cause of death classification system for maternal deaths in low and middle-income countries: results from the Global Network Maternal Newborn Health Registry*^7^ | 2001  2008  2012  2012  2015  2015  2018 | Kilonzo A, Kouletio M, Whitehead SJ, Curtis KM, & McCarthy BJ.  Barnett S, Nair N, Tripathy P, Borghi J, Rath S, & Costello A.  Prata N, Gerdts C, & Gessessew A.  Bowden S, Braker K, Checchi F, & Wong S.  Bayley O, Chapota H, Kainja E, et al.  Yaya Y, Data T, & Lindjørn B.  Pasha O, McClure FM, Saleem S, et al. | Tanzania  India  Ethiopia  Chad  Malawi  Ethiopia  Low- and middle-income countries |
| Direct sisterhood | *Maternal mortality estimation: separating pregnancy-related and non-pregnancy-related risks*^8^  *Adult female mortality: levels and causes*^9^  *How should we measure maternal mortality in the developing world? A comparison of household deaths and sibling history approaches*^10^  *Estimation of maternal and neonatal mortality at the subnational level in Liberia*^11^  *Unmasking inequalities: Sub-national maternal and child mortality data from two urban slums in Lagos, Nigeria tells the story*^12^  *The influence of the social and cultural environment on maternal mortality in Nigeria: Evidence from the 2013 demographic and health survey*^13^  *Evaluating maternal mortality ratios of Nepal with 2003 Nepal world health survey estimates*^14^  *Estimating maternal mortality in remote rural regions: An application of the sisterhood method in Tajikistan*^15^  *Levels and determinants of maternal mortality in northern and southern Nigeria*^16^  *Ratios and determinants of maternal mortality: a comparison of geographic differences in the northern and southern regions of Cameroon*^17^  *Age differentials in pregnancy-related deaths in selected African countries*^18^  *Reduction in maternal mortality ratio varies by district in Sidama Regional State, southern Ethiopia: Estimates by cross-sectional studies using the sisterhood method and a household survey of pregnancy and birth outcomes*^19^  *A comparison of approaches to measuring maternal mortality in Bangladesh, Mozambique, and Bolivia*^20^ | 1995  2001  2006  2014  2017  2017  2018  2019  2019  2020  2021  2022  2022 | Steklov G.  Hill KL, El-Arifeen S, Chowdhury HR, & Ranhman S.  Hill K, El Arifeen S, Koenig M, Al-Sabir A, Jamil K, & Raggers H.  Moseson H, Massaquoi M, Bawo L, et al.  Anastasi E, Enakem E, Hill O, Adebayo Oluwakemi A, Abayomi O, & Bernasconi A.  Ariyo O, Ozodiegwu ID, Doctor HV.  Bhandary S.  Liese KL, Pauls H, Robinson S, &Patil C.  Meh C, Thind A, Ryan B, & Terry A.  Meh C, Thind A, & Terry A.  Izugara C.  Kea A, Lindtjorn B, Tekele A, & Hinderaker S.  Singh K, Li Q, Ahsan KZ, Curtis S, & Weiss W. | Bolivia  Bangladesh  Bangladesh  Liberia  Nigeria  Nigeria  Nepal  Tajikistan  Nigeria  Cameroon  Namibia, Kenya, Sierra Leone  Ethiopia  Bangladesh, Mozambique, Bolivia |
| Hospital- or facility-based | *Implementing a facility-based maternal and perinatal health care surveillance system in Afghanistan*^21^  *Facility-based maternal death reviews: effects on maternal mortality in a district hospital in Senegal*^22^  *Maternal deaths in Sagamu in the new millennium: a facility-based retrospective analysis*^23^  *Facility-based maternal death review in three districts in the central region of Malawi*^24^  *Improving obstetric care in low-resource settings: Implementation of facility-based maternal death reviews in five pilot hospitals in Senegal*^25^  *Maternal mortality in Jos Nigeria: A facility based prospective review*^26^  *Maternal mortality in Ghana: a hospital-based review*^27^  *Facility-based review of 296 maternal deaths at a tertiary centre in India: Could they be prevented?*^28^  *Maternal mortality in central province, Kenya, 2009-2010*^29^  *Facility based maternal death review at tertiary care hospital: A small effort to explore hidden facts*^30^  *Trends in health facility based maternal mortality in Central Region, Kenya: 2008-2012*^31^  *Maternal death audit in Rwanda 2009-2013: a nationwide facility-based retrospective cohort study*^32^  *Maternal death review and surveillance: The case of Central Hospital, Benin City, Nigeria*^33^  *Maternal death reviews: A retrospective case series of 90 hospital-based maternal deaths in 11 hospitals in Indonesia*^34^  *Patterns and causes of hospital maternal mortality in Tanzania: A 10-year retrospective analysis*^35^  *Hospital-based retrospective cross-sectional study to analyse the causes of maternal deaths at a tertiary health care facility*^36^ | 2005  2006  2006  2008  2009  2011  2012  2013  2014  2016  2016  2016  2019  2019  2019  2022 | Dott MM, Orakail N, Ebadi H, et al.  Dumont A, Gaye A, de Bernis L, Chaillet N, Landry A, Delage J, & Bouvier-Colle, M.  Oladapo OT, Lamina MA, & Fakoya TA.  Kongnyuy EJ, Mlava G, & van der Broek N.  Dumont A, Tourigny C, & Fournier P.  Ngwan SD & Swende TZ.  Lee QY, Odoi AT, Opare-Addo H, & Dassah ET.  Goswami D, Rathore AM, Batra S, Dubey C, Tyagi S, & Wadhwa L.  Muchemi OM & Gichogo AW.  Mehta M & Bavarva N.  Muchemi OM, Gichogo AW, Mungai JG, & Roka ZG.  Sayinzoga F, Bijlmakers L, van Dillen J, Mivumbi V, Ngabo F, & van der Velden K.  Aikpitanyi J, Ohenhen V, Ugbodaga P, et al.  Baharuddin M, Amelia D, Suhowatsky S, Kusuma A, Suhargono MH, & Eng B.  Bwana VM, Rumisha SF, Mremi IR, Lyimo EP, & Mboera LEG.  Singh NP, Jain PK, Saxena D, Takhelchangbam ND, & Singh A. | Afghanistan  Senegal  Nigeria  Malawi  Senegal  Nigeria  Ghana  India  Kenya  India  Kenya  Rwanda  Nigeria  Indonesia  Tanzania  India |
| Indirect sisterhood | *Population based estimates of maternal mortality in Mojokerto, East Java (the application of indirect technique: Sisterhood method)*  *Estimating maternal mortality in Djibouti: an application of the sisterhood method*^37^  *Estimating maternal mortality by sisterhood method in rural Zimbabwe*^38^  *Estimating maternal mortality in rural areas of Mexico: The application of an indirect demographic method*^39^  *Assessment of maternal mortality in Tanzania*^40^  *The Bali Indirect maternal mortality study*^41^  *A comparison of sisterhood information on causes of maternal death with the registration causes of maternal death in Matlab, Bangladesh*^42^  *The sisterhood method of estimating maternal mortality: The Matlab experience*^43^  *Applying the sisterhood method for estimating maternal mortality to a health facility-based sample: A comparison with results from a household-based sample*^44^  *Maternal mortality in a Kenyan pastoralist population*^45^  *Direct and indirect estimates of maternal mortality in rural Burkina Faso*^46^  *High maternal mortality levels and additional risk from poor accessibility in two districts of northern province, Zambia*^47^  *Maternal mortality in rural Zambia*^48^  *Maternal mortality among the Kassena-Nankana of northern Ghana*^49^  *Estimates of maternal mortality by the sisterhood method in rural northern Tanzania: A household sample and an antenatal clinic sample*^50^  *Maternal mortality in a rural district of southeastern Tanzania: An application of the sisterhood method*^51^  *Maternal mortality estimated using the sisterhood method in Gulu district, Uganda*^52^  *Maternal mortality in Herat province, Afghanistan, in 2002: An indicator of women's human rights*^53^  *Survey on maternal mortality in Swaziland using the sisterhood method*^54^  *Estimates of maternal mortality in western Tanzania by the sisterhood method*^55^  *The use of the sisterhood method for estimating maternal mortality ratio in Lagos state, Nigeria*^56^  *High maternal mortality estimated by the sisterhood method in a rural area of Mali*^57^  *Repetition of a sisterhood survey at district level in Malawi: The challenge to achieve MDG 5*^58^  *High maternal mortality in rural south-west Ethiopia: Estimate by using the sisterhood method*^59^  *Maternal mortality in northern Nigeria: Findings of a health and demographic surveillance system in Zamfara State, Nigeria*^60^  *Estimating maternal mortality level in rural northern Nigeria by the sisterhood method*^61^  *Place of birth or place of death: An evaluation of 1139 maternal deaths in Nigeria*^62^  *Estimation of maternal mortality using the indirect sisterhood method in Suleja, Niger state-Nigeria*^63^  *Community study of maternal mortality in south west Nigeria: How applicable is the sisterhood method*^64^  *High maternal mortality in Jigawa state, northern Nigeria estimated using the sisterhood method*^65^  *Maternal mortality ratio in selected rural communities in Kebbi state, northwest Nigeria*^66^  *Estimation of maternal mortality by sisterhood method in two rural communities in Kaduna State, Nigeria*^67^  *Estimating maternal mortality in remote rural regions: An application of the sisterhood method in Tajikistan*^15^  *Reduction in maternal mortality ratio varies by district in Sidama regional state, southern Ethiopia: Estimates by cross-sectional studies using the sisterhood method and a household survey of pregnancy and birth outcomes*^19^ | 1989  1991  1993  1994  1994  1994  1995  1995  1996  1996  1997  1997  1997  1999  2000  2000  2000  2002  2002  2003  2011  2011  2011  2012  2012  2012  2013  2013  2013  2017  2018  2019  2019  2022 | Soemantri S.  David P, Kawar S, & Graham W.  Oosterhuis JW.  Hernández B, Chirinos J, Romero M, & Langer A.  Walraven GE, Mkanje RJ, van Roosmalen J, van Dongen PW, & Dolmans WM.  Wirawan DN & Linnan M.  Shahidullah M.  Shahidullah M.  Danel I, Graham W, Stupp P, & Castillo P.  Mace R & Sear R.  Garenne M, Sauerborn R, Nougtara A, Borchert M, Benzler J, & Diesfeld J.  Le Bacq F & Rietsema A.  Vork FC, Kyanamina S, & van Roosmalen J.  Ngom P, Akweongo P, Adongo P, Bawah AA, & Bikna F.  Evjen-Olsen B, Hinderaker S, Kazaura M, Bergsjø P, Gasheka P, & Kvåle G.  Font F, Alonso Gonzalez M, Nathan R, et al.  Orach CG.  Amowitz L, Reis C, & Iacopino V.  Lech MM & Zwane A.  Mbaruku G, Vork F, Vyagusa D, Mwakipiti R, & van Roosmalen J.  Oye-Adeniran, BA, Odeyemi KA, Gbadegesin A, et al.  Aa I, Grove MA, Haugsjå AH, & Hinderaker SG.  Beltman JJ, van den Akker T, Lam E, et al.  Yaya Y & Lindtjørn B.  Doctor HV, Olatunji A, Findley SE, Afenyadu GY, Abdulwahab A, & Jumare A.  Doctor HV, Olatunji A, Findley SE, & Afenyadu GY.  Adegoke AA, Campbell M, Ogundeji MO, Lawoyin T, & Thomson AM.  Aminat ZY, Aliyu A, & Tukur D.  Adegoke AA, Campbell M, Ogundeji MO, Lawoyin T, & Thomson AM.  Sharma V, Brown W, Kainuwa MA, Leight J, & Nyqvist MB.  Gulumbe U, Alabi O, Omisakin OA, & Omoleke S.  Usman NO, Abdullahi HM, Nmadu AG, Omole VN, & Ango JT.  Liese KL, Pauls H, Robinson S, Patil C.  Kea A, Lindtjorn B, Tekele A, Hinderaker S. | Indonesia  Djibouti  Zimbabwe  Mexico  Tanzania  Indonesia  Bangladesh  Bangladesh  Nicaragua  Kenya  Burkina Faso  Zambia  Zambia  Ghana  Tanzania  Tanzania  Uganda  Afghanistan  Eswatini  Tanzania  Nigeria  Mali  Malawi  Ethiopia  Nigeria  Nigeria  Nigeria  Nigeria  Nigeria  Nigeria  Nigeria  Nigeria  Tajikistan  Ethiopia |
| MADE-IN/MADE-FOR | *Using community informants to estimate maternal mortality in a rural district in Pakistan: A feasibility study*^68^  *A nested case-control study of the completeness of maternal death reporting in Banten Province, Indonesia*^69^ | 2015  2020 | Mir AM, Shaikh MS, Qomariyah SN, Rashida G, Khan M, & Masood I.  Qomariyah SN, Sethi R, Izati YN, et al. | Pakistan  Indonesia |
| Motherhood | NA | NA | NA | NA |
| Neighborhood | *A low-cost, community knowledge approach to estimate maternal and jaundice-associated mortality in rural Bangladesh*^70^ | 2018 | Paul RC, Gidding HF, Nazneen A, et al. | Bangladesh |
| Reproductive Age Mortality Study (RAMOS) | *Maternity care in Matlab: Present status and possible interventions*^71^  *Maternal mortality inquiry in a rural community of north India*^72^  *Causes of reproductive age mortality in low socioeconomic settlements of Karachi*^73^  *A community-based nested case-control study of maternal mortality*^74^  *The sisterhood method of estimating maternal mortality: The Matlab experience*^43^  *A comparison of sisterhood information on causes of maternal death with the registration causes of maternal death in Matlab, Bangladesh*^42^  *Maternal mortality in different Pakistani sites:*  *Ratios, clinical causes and determinants*^75^  *A comparison of three verbal autopsy methods to ascertain levels and causes of maternal deaths in Matlab, Bangladesh*^76^  *Deaths among women of reproductive age in Cape Verde, causes and avoidability*^77^  *Who dies from what? Determining cause of death in South Africa's rural north-east*^78^  *Mortality and causes of death in Jordan 1995-96: Assessment by verbal autopsy*^79^  *Validation and application of verbal autopsies in a rural area of South Africa*^80^  *Maternal mortality in Vietnam in 1994-95*^81^  *Maternal mortality among Afghan refugees in Pakistan, 1999-2000*^82^  *Pregnancy-related deaths in rural Rajasthan, India: Exploring causes, context, and care-seeking through verbal autopsy*^83^  *Improving maternal mortality reporting at the community level with a 4-question modified reproductive age mortality survey (RAMOS)*^84^  *Mortality in women of reproductive age in rural South Africa*^85^  *Measuring maternal mortality using a Reproductive Age Mortality Study (RAMOS)*^86^ | 1988  1989  1993  1994  1995  1995  1997  1998  1999  1999  1999  2000  2000  2002  2009  2011  2013  2016 | Fauveau V & Chakraborty J.  Kumar R, Sharma AK, Barik S, & Kumar V.  Fikree FF, Karim MS, Midhet F, & Berendes HW.  Fikree FF, Gray RH, Berendes HW, & Karim MS.  Shahidullah M.  Shahidullah M.  Fikree FF, Midhet F, Sadruddin S, & Berendes H.  Ronsmans C, Vanneste A,  Chakraborty J, & Ginneken J.  Wessel H, Reitmaier P, Dupret A, Rocha E, Cnattingius S, & Bergström S.  Kahn K, Tollman SM, Garenne M, & Gear JSS.  Khoury SA, Massad D, & Fardous T.  Kahn K, Tollman SM, Garenne M, & Gear JSS.  Hanenberg R, Vach T, Vinh D, & Sokal D.  Bartlett LA, Jamieson DJ, Kahn T, Sultana M, Wilson HG, & Duerr A.  Iyengar K, Iyengar SD, Suhalka V, & Dashora K.  Geynisman J, Latimer A, Ofosu A, & Anderson FWJ.  Nabukalu D, Klipstein-Grobusch K, Herbst K, & Newell M.  Mgawadere, F, Unkels R, Adegoke A, & van den Broek Nynke. | Bangladesh  India  Pakistan  Pakistan  Bangladesh  Bangladesh  Pakistan  Bangladesh  Cape Verde  South Africa  Jordan  South Africa  Vietnam  Afghanistan  India  Ghana  South Africa  Malawi |

**References**

1. Kilonzo A, Kouletio M, Whitehead SJ, Curtis KM, McCarthy BJ. Improving surveillance for maternal and perinatal health in 2 districts of rural Tanzania. *Am J Public Health*. 2001;91(10):1636-1640. doi:10.2105/ajph.91.10.1636

2. Barnett S, Nair N, Tripathy P, Borghi J, Rath S, Costello A. A prospective key informant surveillance system to measure maternal mortality - findings from indigenous populations in Jharkhand and Orissa, India. *BMC Pregnancy Childbirth*. 2008;8:6. doi:10.1186/1471-2393-8-6

3. Prata N, Gerdts C, Gessessew A. An innovative approach to measuring maternal mortality at the community level in low-resource settings using mid-level providers: a feasibility study in Tigray, Ethiopia. *Reprod Health Matters*. 2012;20(39):196-204. doi:10.1016/S0968-8080(12)39606-7

4. Bowden S, Braker K, Checchi F, Wong S. Implementation and utilisation of community-based mortality surveillance: A case study from Chad. *Conflict and health*. 2012;6:11. doi:10.1186/1752-1505-6-11

5. Bayley O, Chapota H, Kainja E, et al. Community-linked maternal death review (CLMDR) to measure and prevent maternal mortality: a pilot study in rural Malawi. *BMJ open*. 2015;5(4):e007753.

6. Yaya Y, Data T, Lindtjørn B. Maternal mortality in rural south Ethiopia: outcomes of community-based birth registration by health extension workers. *PLoS One*. 2015;10(3):e0119321. doi:10.1371/journal.pone.0119321

7. Pasha O, McClure EM, Saleem S, et al. A prospective cause of death classification system for maternal deaths in low and middle-income countries: results from the Global Network Maternal Newborn Health Registry. *BJOG*. 2018;125(9):1137-1143. doi:10.1111/1471-0528.15011

8. Stecklov G. Maternal mortality estimation: separating pregnancy-related and non-pregnancy-related risks. *Stud Fam Plann*. 1995;26(1):33-38.

9. Hill KL, El-Arifeen S, Chowdhury HR, Rahman S. Adult female mortality: levels and causes. *Bangladesh maternal health services and maternal mortality survey 2001*. Published online 2001.

10. Hill K, El Arifeen S, Koenig M, Al-Sabir A, Jamil K, Raggers H. How should we measure maternal mortality in the developing world? A comparison of household deaths and sibling history approaches. *Bull World Health Organ*. 2006;84(3):173-180. doi:10.2471/blt.05.027714

11. Moseson H, Massaquoi M, Bawo L, et al. Estimation of maternal and neonatal mortality at the subnational level in Liberia. *Int J Gynaecol Obstet*. 2014;127(2):194-200. doi:10.1016/j.ijgo.2014.05.007

12. Anastasi E, Ekanem E, Hill O, Adebayo Oluwakemi A, Abayomi O, Bernasconi A. Unmasking inequalities: Sub-national maternal and child mortality data from two urban slums in Lagos, Nigeria tells the story. *PLoS One*. 2017;12(5):e0177190. doi:10.1371/journal.pone.0177190

13. Ariyo O, Ozodiegwu ID, Doctor HV. The influence of the social and cultural environment on maternal mortality in Nigeria: Evidence from the 2013 demographic and health survey. *PLoS One*. 2017;12(12):e0190285. doi:10.1371/journal.pone.0190285

14. Bhandary S. Evaluating maternal mortality ratios of Nepal with 2003 Nepal world health survey estimates. *Journal of General Practice and Emergency Medicine of Nepal*. 2018;5(7):15-19.

15. Liese KL, Pauls H, Robinson S, Patil C. Estimating Maternal Mortality in Remote Rural Regions: an Application of the Sisterhood Method in Tajikistan. *Cent Asian J Glob Health*. 2019;8(1):341. doi:10.5195/cajgh.2019.341

16. Meh C, Thind A, Ryan B, Terry A. Levels and determinants of maternal mortality in northern and southern Nigeria. *BMC Pregnancy Childbirth*. 2019;19(1):417. doi:10.1186/s12884-019-2471-8

17. Meh C, Thind A, Terry AL. Ratios and determinants of maternal mortality: a comparison of geographic differences in the northern and southern regions of Cameroon. *BMC Pregnancy Childbirth*. 2020;20(1):194. doi:10.1186/s12884-020-02879-y

18. Izugbara C. Age differentials in pregnancy-related deaths in selected African countries. *J Obstet Gynaecol*. 2021;41(4):516-521. doi:10.1080/01443615.2020.1754367

19. Kea A, Lindtjorn B, Tekele A, Hinderaker S. Reduction in maternal mortality ratio varies by district in Sidama Regional State, southern Ethiopia: Estimates by cross-sectional studies using the sisterhood method and a household survey of pregnancy and birth outcomes. Published online October 4, 2022. doi:10.1101/2022.10.02.22280613

20. Singh K, Li Q, Ahsan KZ, Curtis S, Weiss W. A comparison of approaches to measuring maternal mortality in Bangladesh, Mozambique, and Bolivia. *Popul Health Metr*. 2022;20(1):5. doi:10.1186/s12963-022-00281-8

21. Dott MM, Orakail N, Ebadi H, et al. Implementing a facility-based maternal and perinatal health care surveillance system in Afghanistan. *Journal of midwifery & women’s health*. 2005;50(4):296-300.

22. Dumont A, Gaye A, de Bernis L, et al. Facility-based maternal death reviews: effects on maternal mortality in a district hospital in Senegal. *Bull World Health Organ*. 2006;84(3):218-224. doi:10.2471/blt.05.023903

23. Oladapo OT, Lamina MA, Fakoya TA. Maternal deaths in Sagamu in the new millennium: a facility-based retrospective analysis. *BMC Pregnancy and Childbirth*. 2006;6:1-7.

24. Facility-Based Maternal Death Review In Three Districts In The Central Region of Malawi. ResearchGate. Published October 1, 2008. Accessed April 11, 2023. https://www.researchgate.net/publication/23708457_Facility-Based_Maternal_Death_Review_In_Three_Districts_In_The_Central_Region_of_Malawi

25. Improving obstetric care in low-resource settings: Implementation of facility-based maternal death reviews in five pilot hospitals in Senegal. ResearchGate. Published September 1, 2009. Accessed April 11, 2023. https://www.researchgate.net/publication/26690926_Improving_obstetric_care_in_low-resource_settings_Implementation_of_facility-based_maternal_death_reviews_in_five_pilot_hospitals_in_Senegal

26. Ngwan SD, Swende TZ. Maternal mortality in Jos Nigeria: A facility based prospective review. *Int J Biol Med Res*. 2011;2(2):565-568.

27. Lee QY, Odoi AT, Opare-Addo H, Dassah ET. Maternal mortality in Ghana: a hospital-based review. *Acta Obstet Gynecol Scand*. 2012;91(1):87-92. doi:10.1111/j.1600-0412.2011.01249.x

28. Goswami D, Rathore AM, Batra S, Dubey C, Tyagi S, Wadhwa L. Facility-based review of 296 maternal deaths at a tertiary centre in I ndia: Could they be prevented? *Journal of Obstetrics and Gynaecology Research*. 2013;39(12):1569-1579.

29. Muchemi OM, Gichogo AW. Maternal mortality in central province, Kenya, 2009-2010. *The Pan African medical journal*. 2014;17.

30. Mehta M, Bavarva N. Facility Based Maternal Death Review at Tertiary Care Hospital: A Small Effort to Explore Hidden Facts. *Appl Med Res 2016; 1 (4): 126*. 2016;9.

31. Muchemi OM, Gichogo AW, Mungai JG, Roka ZG. Trends in health facility based maternal mortality in Central Region, Kenya: 2008-2012. *Pan Afr Med J*. 2016;23:259. doi:10.11604/pamj.2016.23.259.8262

32. Sayinzoga F, Bijlmakers L, van Dillen J, Mivumbi V, Ngabo F, van der Velden K. Maternal death audit in Rwanda 2009-2013: a nationwide facility-based retrospective cohort study. *BMJ Open*. 2016;6(1):e009734. doi:10.1136/bmjopen-2015-009734

33. Aikpitanyi J, Ohenhen V, Ugbodaga P, et al. Maternal death review and surveillance: The case of Central Hospital, Benin City, Nigeria. *PLoS One*. 2019;14(12):e0226075. doi:10.1371/journal.pone.0226075

34. Baharuddin M, Amelia D, Suhowatsky S, Kusuma A, Suhargono MH, Eng B. Maternal death reviews: A retrospective case series of 90 hospital-based maternal deaths in 11 hospitals in Indonesia. *International Journal of Gynecology & Obstetrics*. 2019;144:59-64.

35. Bwana VM, Rumisha SF, Mremi IR, Lyimo EP, Mboera LEG. Patterns and causes of hospital maternal mortality in Tanzania: A 10-year retrospective analysis. *PLoS One*. 2019;14(4):e0214807. doi:10.1371/journal.pone.0214807

36. Singh NP, Jain PK, Saxena D, Takhelchangbam ND, Singh A. Hospital-Based Retrospective Cross-sectional Study to Analyse the Causes of Maternal Deaths at a Tertiary Health Care Facility. *J Family Med Prim Care*. 2022;11(8):4603-4609. doi:10.4103/jfmpc.jfmpc_1551_21

37. David P, Kawar S, Graham W. Estimating maternal mortality in Djibouti: an application of the sisterhood method. *Int J Epidemiol*. 1991;20(2):551-557. doi:10.1093/ije/20.2.551

38. Oosterhuis JW. Estimating maternal mortality by sisterhood method in rural Zimbabwe. *Trop Doct*. 1993;23(2):67-68. doi:10.1177/004947559302300210

39. Hernández B, Chirinos J, Romero M, Langer A. Estimating maternal mortality in rural areas of Mexico: the application of an indirect demographic method. *Int J Gynaecol Obstet*. 1994;46(3):285-289. doi:10.1016/0020-7292(94)90407-3

40. Walraven GE, Mkanje RJ, van Roosmalen J, van Dongen PW, Dolmans WM. Assessment of maternal mortality in Tanzania. *Br J Obstet Gynaecol*. 1994;101(5):414-417. doi:10.1111/j.1471-0528.1994.tb11914.x

41. Wirawan DN, Linnan M. The Bali Indirect Maternal Mortality Study. *Stud Fam Plann*. 1994;25(5):304-309.

42. Shahidullah M. A comparison of sisterhood information on causes of maternal death with the registration causes of maternal death in Matlab, Bangladesh. *Int J Epidemiol*. 1995;24(5):937-942. doi:10.1093/ije/24.5.937

43. Shahidullah M. The sisterhood method of estimating maternal mortality: the Matlab experience. *Stud Fam Plann*. 1995;26(2):101-106.

44. Danel I, Graham W, Stupp P, Castillo P. Applying the sisterhood method for estimating maternal mortality to a health facility-based sample: a comparison with results from a household-based sample. *Int J Epidemiol*. 1996;25(5):1017-1022. doi:10.1093/ije/25.5.1017

45. Mace R, Sear R. Maternal mortality in a Kenyan pastoralist population. *Int J Gynaecol Obstet*. 1996;54(2):137-141. doi:10.1016/0020-7292(96)02691-4

46. Garenne M, Sauerborn R, Nougtara A, Borchert M, Benzler J, Diesfeld J. Direct and indirect estimates of maternal mortality in rural Burkina Faso. *Stud Fam Plann*. 1997;28(1):54-61.

47. Le Bacq F, Rietsema A. High maternal mortality levels and additional risk from poor accessibility in two districts of northern province, Zambia. *Int J Epidemiol*. 1997;26(2):357-363. doi:10.1093/ije/26.2.357

48. Vork FC, Kyanamina S, van Roosmalen J. Maternal mortality in rural Zambia. *Acta Obstet Gynecol Scand*. 1997;76(7):646-650. doi:10.3109/00016349709024604

49. Ngom P, Akweongo P, Adongo P, Bawah AA, Binka F. Maternal mortality among the Kassena-Nankana of northern Ghana. *Stud Fam Plann*. 1999;30(2):142-147. doi:10.1111/j.1728-4465.1999.00142.x

50. Evjen-Olsen B, Hinderaker S, Kazaura M, Bergsjø P, Gasheka P, Kvåle G. Estimates of maternal mortality by the sisterhood method in rural northern Tanzania: A household sample and an antenatal clinic sample. *BJOG : an international journal of obstetrics and gynaecology*. 2000;107:1290-1297.

51. Font F, Alonso González M, Nathan R, et al. Maternal mortality in a rural district of southeastern Tanzania: an application of the sisterhood method. *Int J Epidemiol*. 2000;29(1):107-112. doi:10.1093/ije/29.1.107

52. Orach CG. Maternal mortality estimated using the Sisterhood method in Gulu district, Uganda. *Trop Doct*. 2000;30(2):72-74. doi:10.1177/004947550003000205

53. Amowitz L, Reis C, Iacopino V. Maternal Mortality in Herat Province, Afghanistan, in 2002: An Indicator of Women’s Human Rights. *JAMA : the journal of the American Medical Association*. 2002;288:1284-1291. doi:10.1001/jama.288.10.1284

54. Lech MM, Zwane A. Survey on maternal mortality in Swaziland using the sisterhood method. *Paediatr Perinat Epidemiol*. 2002;16(2):101-107. doi:10.1046/j.1365-3016.2002.00411.x

55. Mbaruku G, Vork F, Vyagusa D, Mwakipiti R, van Roosmalen J. Estimates of maternal mortality in western Tanzania by the sisterhood method. *Afr J Reprod Health*. 2003;7(3):84-91.

56. Oye-Adeniran BA, Odeyemi KA, Gbadegesin A, et al. The use of the sisterhood method for estimating maternal mortality ratio in Lagos state, Nigeria. *J Obstet Gynaecol*. 2011;31(4):315-319. doi:10.3109/01443615.2011.561381

57. Aa I, Grove MA, Haugsjå AH, Hinderaker SG. High maternal mortality estimated by the sisterhood method in a rural area of Mali. *BMC Pregnancy Childbirth*. 2011;11:56. doi:10.1186/1471-2393-11-56

58. Beltman JJ, van den Akker T, Lam E, et al. Repetition of a sisterhood survey at district level in Malawi: the challenge to achieve MDG 5. *BMJ Open*. 2011;1(1):e000080. doi:10.1136/bmjopen-2011-000080

59. Yaya Y, Lindtjørn B. High maternal mortality in rural south-west Ethiopia: estimate by using the sisterhood method. *BMC Pregnancy Childbirth*. 2012;12:136. doi:10.1186/1471-2393-12-136

60. Doctor HV, Olatunji A, Findley SE, Afenyadu GY, Abdulwahab A, Jumare A. Maternal mortality in northern Nigeria: findings of a health and demographic surveillance system in Zamfara State, Nigeria. *Trop Doct*. 2012;42(3):140-143. doi:10.1258/td.2012.120062

61. Doctor H, Findley S, Afenyadu G. Estimating Maternal Mortality Level in Rural Northern Nigeria by the Sisterhood Method. *International Journal of Population Research*. 2012;464657. doi:10.1155/2012/464657

62. Adegoke AA, Campbell M, Ogundeji MO, Lawoyin T, Thomson AM. Place of birth or place of death: an evaluation of 1139 maternal deaths in Nigeria. *Midwifery*. 2013;29(11):e115-121. doi:10.1016/j.midw.2012.11.018

63. Aminat ZY, Aliyu A, Tukur D. Estimation of maternal mortality using the indirect sisterhood method in suleja, Niger state-Nigeria. *Journal of Medicine and Biomedical Research*. 2013;12:131-138.

64. Adegoke AA, Campbell M, Ogundeji MO, Lawoyin TO, Thomson AM. Community Study of maternal mortality in South West Nigeria: how applicable is the sisterhood method. *Matern Child Health J*. 2013;17(2):319-329. doi:10.1007/s10995-012-0977-z

65. Sharma V, Brown W, Kainuwa MA, Leight J, Nyqvist MB. High maternal mortality in Jigawa State, Northern Nigeria estimated using the sisterhood method. *BMC Pregnancy Childbirth*. 2017;17(1):163. doi:10.1186/s12884-017-1341-5

66. Gulumbe U, Alabi O, Omisakin OA, Omoleke S. Maternal mortality ratio in selected rural communities in Kebbi State, Northwest Nigeria. *BMC Pregnancy Childbirth*. 2018;18(1):503. doi:10.1186/s12884-018-2125-2

67. Usman NO, Abdullahi HM, Nmadu AG, Omole VN, Ango JT. Estimation of maternal mortality by sisterhood method in two rural communities in Kaduna State, Nigeria. *Journal of Medicine in the Tropics*. 2019;21(2):62.

68. Mir AM, Shaikh MS, Qomariyah SN, Rashida G, Khan M, Masood I. Using Community Informants to Estimate Maternal Mortality in a Rural District in Pakistan: A Feasibility Study. *Journal of Pregnancy*. 2015;2015:e267923. doi:10.1155/2015/267923

69. Qomariyah SN, Sethi R, Izati YN, et al. No one data source captures all: A nested case-control study of the completeness of maternal death reporting in Banten Province, Indonesia. *PLoS One*. 2020;15(5):e0232080. doi:10.1371/journal.pone.0232080

70. Paul RC, Gidding HF, Nazneen A, et al. A Low-Cost, Community Knowledge Approach to Estimate Maternal and Jaundice-Associated Mortality in Rural Bangladesh. *Am J Trop Med Hyg*. 2018;99(6):1633-1638. doi:10.4269/ajtmh.17-0974

71. Fauveau V, Chakraborty J. Maternity care in Matlab: Present status and possible interventions. Published online 1988.

72. Kumar R, Sharma AK, Barik S, Kumar V. Maternal mortality inquiry in a rural community of north India. *International Journal of Gynecology & Obstetrics*. 1989;29(4):313-319.

73. Fikree FF, Karim MS, Midhet F, Berendes HW. Causes of reproductive age mortality in low socioeconomic settlements of Karachi. *Journal of Pakistan Medical Association*. 1993;43(10):208.

74. Fikree FF, Gray RH, Berendes HW, Karim MS. A community-based nested case-control study of maternal mortality. *International Journal of Gynecology & Obstetrics*. 1994;47(3):247-255.

75. Fikree F, Midhet F, Sadruddin S, Berendes H. Maternal mortality in different Pakistani sites: Ratios, clinical causes and determinants. *Acta obstetricia et gynecologica Scandinavica*. 1997;76:637-645. doi:10.3109/00016349709024603

76. Ronsmans C, Vanneste A, Chakraborty J, Ginneken J. A comparison of three verbal autopsy methods to ascertain levels and causes of maternal deaths in Matlab, Bangladesh. *International journal of epidemiology*. 1998;27:660-666. doi:10.1093/ije/27.4.660

77. Wessel H, Reitmaier P, Dupret A, Rocha E, Cnattingius S, Bergström S. Deaths among women of reproductive age in Cape Verde, causes and avoidability. *Acta Obstetricia et Gynecologica Scandinavica*. 1999;78(3):225-232.

78. Kahn K, Tollman SM, Garenne M, Gear JS. Who dies from what? Determining cause of death in South Africa’s rural north-east. *Tropical Medicine & International Health*. 1999;4(6):433-441.

79. Khoury SA, Massad D, Fardous T. Mortality and causes of death in Jordan 1995-96: assessment by verbal autopsy. *Bulletin of the World Health Organization*. 1999;77(8):641.

80. Kahn K, Tollman S, Garenne M, Gear J. Validation and application of verbal autopsies in a rural area of South Africa | Request PDF. *Tropical Medicine & International Health*. Published online 2000. Accessed April 7, 2023. https://www.researchgate.net/publication/12204685_Validation_and_application_of_verbal_autopsies_in_a_rural_area_of_South_Africa

81. Hanenberg R, Vach T, Vinh D, Sokal D. Maternal mortality in Vietnam in 1994-95. *Studies in family planning*. 2000;30:329-338.

82. Bartlett LA, Jamieson DJ, Kahn T, Sultana M, Wilson HG, Duerr A. Maternal mortality among Afghan refugees in Pakistan, 1999-2000. *The Lancet*. 2002;359(9307):643-649.

83. Iyengar K, Iyengar SD, Suhalka V, Dashora K. Pregnancy-related deaths in rural Rajasthan, India: exploring causes, context, and care-seeking through verbal autopsy. *Journal of health, population, and nutrition*. 2009;27(2):293.

84. Geynisman J, Latimer A, Ofosu A, Anderson FWJ. Improving maternal mortality reporting at the community level with a 4-question modified reproductive age mortality survey (RAMOS). *Int J Gynaecol Obstet*. 2011;114(1):29-32. doi:10.1016/j.ijgo.2011.01.011

85. Nabukalu D, Klipstein-Grobusch K, Herbst K, Newell ML. Mortality in women of reproductive age in rural South Africa. *Global Health Action*. 2013;6(1):22834.

86. Mgawadere F, Unkels R, Adegoke A, van den Broek N. Measuring maternal mortality using a Reproductive Age Mortality Study (RAMOS). *BMC Pregnancy and Childbirth*. 2016;16(1):291. doi:10.1186/s12884-016-1084-8
