# Supplementary material for: Maternal mortality estimation methodologies: a scoping review and evaluation of suitability for use in humanitarian settings
Source: Confl Health. 2024 Dec 19;18:75. doi: 10.1186/s13031-024-00636-y (PMC11657123; doi:10.1186/s13031-024-00636-y)
Supplement: Supplementary file 2 — Additional file 2. MADE-IN/MADE-FOR methodology completed evaluation form. Additional file 2 shows the completed evaluation form for the MADE-IN/MADE-FOR methodology. [file 13031_2024_636_MOESM2_ESM.docx]

**Additional file 2. MADE-IN/MADE-FOR methodology completed evaluation form**

| **Category** | **The MADE-IN/MADE-FOR [Maternal Deaths from Informants/Maternal Death Follow on Review] method (Qomariyah, et al., 2010)**^1^ | | |
| --- | --- | --- | --- |
|  | **Notes from original implementation** | **Notes from additional implementations** | **Score (1-4)** |
| *Summary of methodology* | MADE-IN   - Kaders (health volunteers) and/or head of neighborhood units (community level volunteers) conducted listing-meetings of village informants to list details of deaths of WRA in a village - If both kader and head of neighborhood unit's network used in a village, two separate meetings - Information, instructions, and forms distributed to village informants before meetings for WRA deaths in previous two years - Timing of death in relation to any pregnancy, age of woman, date of death, residence of woman, name, and address of a relative - Informants asked to bring completed forms to meeting, study and forms explained verbally, questions answered, opportunity given for informants to correct forms in light of improved understanding - Meeting discussed all deaths listed by informants and collectively agreed on a "consolidated" list of deaths and pregnancy-related deaths, also included a visit to village informants who did not attend meetings   MADE-FOR   - Visits to named relative of likely pregnancy-related deaths on a consolidated MADE-in listing meeting list - To confirm details on form for "eligible" deaths, i.e., time period and district, additional info about circumstances and cause of death through structured verbal autopsy - Allowed estimation of cause of death using a computerized algorithm (InterVA-M) - Socio-economic info and use of health services near time of death also recorded - MADE-FOR provided village level estimates of number of deaths of WRA: pregnancy-related deaths | | |
| *Data sources* | Community informants at meetings, comparison between two sets of informants, verbal autopsy | - Four networks to provide information: Lady Health Network, religious leaders (Imams), elected councilors, nikah registrants (record marriages); secretary of the union council (local government) invited informants to preparatory meetings; two separate meetings -- preparatory and listing for each group separately; MADE FOR: used WHO VA tool for all household with a death^2^ - Kaders and heads of neighborhood units^3^ | **3** |
| *Definitions* | - Used pregnancy-related death definition - Death of a woman while pregnant or within 42 days of termination of pregnancy, irrespective of COD - Found that 97.5% of pregnancy-related death were from maternal causes, so does not matter whether definition is pregnancy-related death or maternal death | - All deaths to women 12-50 years; whether woman died during pregnancy, delivery, within 6 weeks postpartum and abortion (i.e., WHO), date and place of death, age, name of husband, residential address^2^ | **3** |
| *Sample size* | At least one death | - At least one death^2^ | **4** |
| *Timing of point estimate relative to data collection* | Collected data on prior two years | - Collected data for the prior two years^2^ - Collected data for one calendar year^3^ | **3** |
| *Bias* | - Non-response bias (informant at meeting or available for follow up survey) - Recall bias (whether someone was pregnant, whether they died in the time period, cause of death) - Selection bias (miss early deaths [i.e., before others know about pregnancy]) - Selection bias (assumes that someone tells others about pregnancy) - Selection bias (assumes that the individual that died has [living] siblings AND that they were present at death AND can provide info about circumstances of death) - Assumptions from capture-recapture:   - Set of individuals or events is fixed, i.e., there is a closed population   - Individuals captured by RT and kader can be matched (i.e., deaths can be matched and not double counted)   - Capture in the second sample is independent of the capture in the first (contamination)   - Within each database, probability of capture does not differ between individuals (certain types of deaths not reported by either network, like early pregnancy deaths) |  | **2.5** |
|  |  |  |  |
| *Human resources* | - 18 weeks: 436 person-weeks for all villages in two districts - 24 people a week for 18 weeks - 708 villages in Serang and Pandeglang Districts: 38 sub district health centers for 1.9 million people in Serand and 1.1 in Pandeglang | NA | **2** |
| *Time needed for implementation* | - 18 weeks: 436 person-weeks for all villages in two districts - 24 people a week for 18 weeks | NA | **2.5** |
| *Data collection training* | See *Summary of methodology* above | NA | **3.5** |
| *Statistical training* | Allowed estimation of cause of death using a computerized algorithm (InterVA-M) | NA | **3** |
| *Digitalization* | Already digitized in InterVA-M | Data collected in EPI Info^3^ | **4** |
| *Cost* | - The number of pregnancy-related deaths identified per “survey effort” (data collector-weeks) is one measure used to compare MADE-IN/MADE-FOR with other methods for identifying maternal deaths. Another measure used is “cost per woman-year of exposure.” Exposure is calculated from the number of WRA in the area, multiplied by the number of years of exposure. Costs were divided between MADE-IN or MADE-FOR, either as they fall clearly to one (such as informants’ transportation fee, which is a MADE-IN expenditure), or, for more general expenditure (wages, accommodation for the survey team), based on the number of data-collector-days spent on MADE-IN and MADE-FOR, which was generally similar. Information on the cost of population survey, of which the estimate of live births is used, is illustrated as unit cost for capturing a live birth. - Total study cost US$154,271: salaries for field workers, travel cost, supplies, services, administration, and capital Informants given transportation fee to meeting and light meal Cost of population survey: US$130,435 - MADE-FOR increased costs by about 50%, but prevented false positives and underestimates from kader network - Cost per woman-year risk of exposure: MADE-IN in US$0.056, MADE-FOR US$0.046, Total, US$0.102 | Per-unit WRA in the study area was US$0.12^2^ | **3** |
| *Total score* | | | **33.5/44** |

**References**

1. Gerdts C, Prata N, Gessessew A. An unequal burden: risk factors for severe complications following unsafe abortion in Tigray, Ethiopia. *Int J Gynaecol Obstet*. 2012;118 Suppl 2:S107-112. doi:10.1016/S0020-7292(12)60008-3

2. Mir AM, Shaikh MS, Qomariyah SN, Rashida G, Khan M, Masood I. Using Community Informants to Estimate Maternal Mortality in a Rural District in Pakistan: A Feasibility Study. *Journal of Pregnancy*. 2015;2015:e267923. doi:10.1155/2015/267923

3. Qomariyah SN, Sethi R, Izati YN, et al. No one data source captures all: A nested case-control study of the completeness of maternal death reporting in Banten Province, Indonesia. *PLoS One*. 2020;15(5):e0232080. doi:10.1371/journal.pone.0232080
