# Supplementary material for: Maternal mortality estimation methodologies: a scoping review and evaluation of suitability for use in humanitarian settings
Source: Confl Health. 2024 Dec 19;18:75. doi: 10.1186/s13031-024-00636-y (PMC11657123; doi:10.1186/s13031-024-00636-y)
Supplement: Supplementary file 3 — Additional file 3. Hospital- or facility-based methodology completed evaluation form. Additional file 3 shows the completed evaluation form for the hospital- or facility-based methodology. [file 13031_2024_636_MOESM3_ESM.docx]

**Additional file 3. Hospital- or facility-based methodology completed evaluation form**

| **Category** | **Hospital- or facility-based record review method (no original)** | | |
| --- | --- | --- | --- |
|  | **Notes from original implementation** | **Notes from additional implementations** | **Score (1-4)** |
| *Summary of methodology* | Review of deaths at facilities, including or not including maternal death review committees; often used to evaluate programs aimed at decreasing maternal mortality | | |
| *Data sources* | NA | - Tertiary care center and referral unit with approximately 6,500 deliveries per year; included interviews with healthcare workers involved^1^ - Nine hospitals; used maternal death review meetings in each hospital where a maternal death occurred within 1-2 months after the maternal death; review meetings were attended by nurses, midwives, doctors working in maternity and public health nurses, laboratory staff, and administrative staff (including senior management) (10-20 people per meeting); before meetings, collected circumstantial information from hospital registers, referral letters, medical records, and interviews with family members; Maternal Death Review Form filled out during the Maternal Death Review by one nominated individual; summary at end of meeting to ensure everyone was in agreement^2^ - District hospital^3^ - Medical College and associated hospital; collected deaths from obstetrics ward actively and every three months from other wards; deaths were presented in quarterly Maternal Death Review meetings where case records were reviewed by the committee members^4^ - Hospital-based retrospective cross-sectional study at a tertiary health care facility; data collected from Facility Based Maternal Death Review Forms maintained by the office of the Chief Medical Officer^5^ - Large woman's hospital^6^ - Referral hospitals (primary-level referral hospitals and regional/teaching hospitals) and data collected from focus group discussions, participant observations of audit meetings, audit documents, and interviews with maternity unit staff; multidisciplinary audit committee with doctors, midwives, nurses, and managers; two data collection forms^7^ - Maternal death audits of all deaths in government-owned, privately-owned, and church-owned hospitals with all health staff who provided care to the decedent at a particular health facility; excluded deaths that were not subjected to a local audit^8^ - Central hospital, the main secondary referral hospital; MPDSR held bimonthly meetings and reviewed consecutive maternal deaths in the hospital; case notes and associated information were retrieved as soon as the deaths occurred and were preserved privately until review by the committees^9^ - Retrospective review of records where the Expanding Maternal and Neonatal Survival program was implemented; extracted de-identified information from patient medical records using a data collection form; each case was assigned a code number by a data analyst who entered the data into a spreadsheet; extracted data reviewed by an expert panel of 24 specialists; four subgroups reviewed 28 cases; cases further reviewed^10^ - Retrospective analysis of all maternal deaths at a university teaching hospital; pulled information from admission and discharge registers, labor and delivery records, and case files from each hospital; cause of death assigned at monthly maternal mortality audit meetings, which were documented in patient case files^11^ - Retrospective review of deaths from July 2009 to June 2010; pulled data from the Maternal Death Notification and Review forms^12^ - Retrospective review based on data from obstetrics, labor, and in-patient notes from teaching hospital, which is a tertiary referral center; maternal death audit team and biostatistics unit identified maternal deaths^13^ - Prospective study of all maternal deaths at a teaching hospital that acts as a referral institute; discussed circumstances of each death at departmental meetings and consensus reached about cause of death, post-mortem for only one patient^14^ - Public owned hospitals from all levels of the healthcare system; extracted from paper resources; thorough search and compilation of all identified forms to record mortality^15^ - Retrospective study of deaths at facilities; retrieved data from paper- and electronic-based notifications of maternal death notification and review forms, monthly district reproductive health reports, and annual reports^16^ | **1** |
| *Definitions* | NA | - Included all registered, emergency, and unregistered pregnant women who admitted into the hospital and died; included interviews with healthcare workers involved^1^ - Any individual that died during pregnancy after being admitted to the hospital, whether in the original department or after transfer to another department; 1976 International Congress of Obstetrician and Gynecologists' definition of death during pregnancy or within 42 days of termination of pregnancy^2^ - All pregnant individuals that were admitted to the district hospital for childbirth; and women referred from other health facilities if the admission was within 24 hours of delivery; women transferred from the study site to other hospitals and obtained information with 6 weeks of transfer^3^ - Deaths while pregnant or within 42 days of termination, from any causes related to pregnancy^7^ - Maternal death according to ICD-10^4,5,8,10,14,15^ - Antepartum, intrapartum, and postpartum/neonatal periods^6^ - Only deaths that occurred at the facility^9^ - Individuals who gave birth at facilities during the study period; used ICD-10 definition^12,16^ - Deaths among those admitted to the hospital; ICD-10^13^ - All records of causes of deaths due to pregnancy and delivery complications among WRA^15^ | **2** |
| *Sample size* | NA | - At least one death:   - 60 deaths^1^   - 43 deaths^2^   - 153 deaths^3^   - 308 deaths^4^   - 181 deaths^5^   - 28 deaths^6^   - 105 deaths^7^   - 987 deaths^8^   - 18 deaths^9^   - 112 deaths^10^   - 75 deaths^11^   - 111 deaths^12^   - 332 deaths^13^   - 56 deaths^14^   - 40,052 deaths to WRA, of which 1,987 were determined to be maternal mortality^15^   - 421 deaths^16^ | **4** |
| *Timing of point estimate relative to data collection* | NA | - No minimum time period - Retrospective:   - Study reviewed deaths from January 2010 to September 2014: 3 years, 9 months^1^   - Study reviewed deaths from January 2007 to December 2007: 1 year^2^   - Study reviewed deaths from April 2005 to March 2010: 5 years^4^   - Study reviewed deaths from May 2004 to June 2005: 1 year, 2 months^7^   - Study reviewed deaths from January 2009 - December 2013: 4 years^8^   - Study reviewed deaths from October 2017 to May 2019: 1 year, 8 months^9^   - Study reviewed deaths from January 2014 - June 2014: 6 months^10^   - Study reviewed deaths from January 2000 to June 2005: 4 years, 6 months^11^   - Study reviewed deaths from January 2008 to June 2010: 1 year, 6 months^13^   - Study reviewed deaths from 2006 to 2015: 10 years^15^   - Study reviewed deaths from July 2008 to June 2012: 4 years^16^ - Prospective:   - Conducted an observational study for 12 months, then implemented a program for 3 years^3^   - Conducted an observational study from June 2006 to May 2008: 2 years^14^ | **3** |
| *Bias* | NA | - Less recall bias when using a prospective study design and data collection^6,14^ - Selection bias:   - Only includes individuals admitted into the hospital, i.e., excludes individuals not admitted to the hospital^1–3,9,10^   - Only includes individuals who gave birth at the facility, i.e., excludes other deaths during pregnancy, in the postpartum period, and among individuals who did not give birth at the facility^12,16^   - Sampled facilities for national representation based on region and contribution to national population (high, medium, low) and then for epidemiological burden and spatial variations of malaria and HIV/AIDS endemicity, patterns of child mortality and human resource coverage^15^ | **1** |
|  |  |  |  |
| *Human resources* | NA | - Review meetings were attended by nurses, midwives, doctors working in maternity and public health nurses, laboratory staff, and administrative staff (including senior management) (10-20 people per meeting)^2^ - Team of general practitioners and midwives; data analyst; expert panel review of 24 specialists^10^ - Biostatistics team and maternal death audit team^13^ - Two research scientists and four data collectors; hospital staff oriented to assist^15^ | **3.5** |
| *Time needed for implementation* | NA | - December 2012: 1 month^12^ - July 2016 to December 2016: 6 months^15^ | **3** |
| *Data collection training* | NA | - Maternal Death Review Form training, but time not reported^2^ - One week of training on implementation of the BABIES program^6^ - Research team and data collectors trained on use of tools; hospital staff and members of medical records unit oriented to the project^15^ | **4** |
| *Statistical training* | NA | Simple calculation because the denominator is the total number of deaths^1–16^ | **4** |
| *Digitalization* | NA | Easy to digitalize | **4** |
| *Cost* | NA | Not reported by any studies | **4** |
| *Total score* | | | **33.5/44** |

**References**

1. Mehta M, Bavarva N. Facility Based Maternal Death Review at Tertiary Care Hospital: A Small Effort to Explore Hidden Facts. *Appl Med Res 2016; 1 (4): 126*. 2016;9.

2. Facility-Based Maternal Death Review In Three Districts In The Central Region of Malawi. ResearchGate. Published October 1, 2008. Accessed April 11, 2023. https://www.researchgate.net/publication/23708457_Facility-Based_Maternal_Death_Review_In_Three_Districts_In_The_Central_Region_of_Malawi

3. Dumont A, Gaye A, de Bernis L, et al. Facility-based maternal death reviews: effects on maternal mortality in a district hospital in Senegal. *Bull World Health Organ*. 2006;84(3):218-224. doi:10.2471/blt.05.023903

4. Goswami D, Rathore AM, Batra S, Dubey C, Tyagi S, Wadhwa L. Facility-based review of 296 maternal deaths at a tertiary centre in I ndia: Could they be prevented? *Journal of Obstetrics and Gynaecology Research*. 2013;39(12):1569-1579.

5. Singh NP, Jain PK, Saxena D, Takhelchangbam ND, Singh A. Hospital-Based Retrospective Cross-sectional Study to Analyse the Causes of Maternal Deaths at a Tertiary Health Care Facility. *J Family Med Prim Care*. 2022;11(8):4603-4609. doi:10.4103/jfmpc.jfmpc_1551_21

6. Dott MM, Orakail N, Ebadi H, et al. Implementing a facility-based maternal and perinatal health care surveillance system in Afghanistan. *Journal of midwifery & women’s health*. 2005;50(4):296-300.

7. Improving obstetric care in low-resource settings: Implementation of facility-based maternal death reviews in five pilot hospitals in Senegal. ResearchGate. Published September 1, 2009. Accessed April 11, 2023. https://www.researchgate.net/publication/26690926_Improving_obstetric_care_in_low-resource_settings_Implementation_of_facility-based_maternal_death_reviews_in_five_pilot_hospitals_in_Senegal

8. Sayinzoga F, Bijlmakers L, van Dillen J, Mivumbi V, Ngabo F, van der Velden K. Maternal death audit in Rwanda 2009-2013: a nationwide facility-based retrospective cohort study. *BMJ Open*. 2016;6(1):e009734. doi:10.1136/bmjopen-2015-009734

9. Aikpitanyi J, Ohenhen V, Ugbodaga P, et al. Maternal death review and surveillance: The case of Central Hospital, Benin City, Nigeria. *PLoS One*. 2019;14(12):e0226075. doi:10.1371/journal.pone.0226075

10. Baharuddin M, Amelia D, Suhowatsky S, Kusuma A, Suhargono MH, Eng B. Maternal death reviews: A retrospective case series of 90 hospital-based maternal deaths in 11 hospitals in Indonesia. *International Journal of Gynecology & Obstetrics*. 2019;144:59-64.

11. Oladapo OT, Lamina MA, Fakoya TA. Maternal deaths in Sagamu in the new millennium: a facility-based retrospective analysis. *BMC Pregnancy and Childbirth*. 2006;6:1-7.

12. Muchemi OM, Gichogo AW. Maternal mortality in central province, Kenya, 2009-2010. *The Pan African medical journal*. 2014;17.

13. Lee QY, Odoi AT, Opare-Addo H, Dassah ET. Maternal mortality in Ghana: a hospital-based review. *Acta Obstet Gynecol Scand*. 2012;91(1):87-92. doi:10.1111/j.1600-0412.2011.01249.x

14. Ngwan SD, Swende TZ. Maternal mortality in Jos Nigeria: A facility based prospective review. *Int J Biol Med Res*. 2011;2(2):565-568.

15. Bwana VM, Rumisha SF, Mremi IR, Lyimo EP, Mboera LEG. Patterns and causes of hospital maternal mortality in Tanzania: A 10-year retrospective analysis. *PLoS One*. 2019;14(4):e0214807. doi:10.1371/journal.pone.0214807

16. Muchemi OM, Gichogo AW, Mungai JG, Roka ZG. Trends in health facility based maternal mortality in Central Region, Kenya: 2008-2012. *Pan Afr Med J*. 2016;23:259. doi:10.11604/pamj.2016.23.259.8262
