# Supplementary material for: Maternal mortality estimation methodologies: a scoping review and evaluation of suitability for use in humanitarian settings
Source: Confl Health. 2024 Dec 19;18:75. doi: 10.1186/s13031-024-00636-y (PMC11657123; doi:10.1186/s13031-024-00636-y)
Supplement: Supplementary file 4 — Additional file 4. Community informant-based methodology completed evaluation form. Additional file 4 shows the completed evaluation form for the community informant-based methodology. [file 13031_2024_636_MOESM4_ESM.docx]

**Additional file 4. Community informant-based methodology completed evaluation form**

| **Category** | **Community informant-based methodology** | | |
| --- | --- | --- | --- |
|  | **Notes from original implementation** | **Notes from additional implementations** | **Score (1-4)** |
| *Summary of methodology* | Use of community-based informants (providers at different levels, government officials, religious leaders) and sometimes facility-based data to identify all deaths and births in a specific catchment area. | | |
| *Data sources* | NA | - Prospective, active surveillance system developed to track pregnancies and birth outcomes in rural or semi-urban settings in several countries; women who provide consent in the catchment areas are followed with outcomes at delivery and 42 days postpartum; registry administrators (nurses or healthcare workers trained on the study) provide surveillance and cause of death is assigned by the healthcare provider for all maternal deaths^1^ - Identification of deaths and births to women of reproductive age (mostly by traditional birth attendants); one key informant per 250 households; key informants met with an interviewee 2/3 times/month to consolidate information and the interviewer verified information by visiting relevant households; a second interviewer visited each household to learn about conditions of death; supervisor conducted verbal autopsy with a family member and with a provider; obstetrician assigned cause of death^2^ - Meeting before project with all levels of the research team and community leaders, community members, and religious leaders; priests, traditional birth attendants, and community-based reproductive health agents were responsible for locating and reporting all births and deaths in their designated areas and helped mid-level providers in locating key informants for verbal autopsy and verification of births and stillbirths; community-based health workers reported to the local health post and once a month, each health post compiled deaths of individuals for verbal autopsy and verification by mid-level providers; one trained nurse or nurse-midwife performed all autopsies; also audited all deaths in health facilities^3^ - Woman dies (unclear how this reported); volunteer and health worker interview the family; then a community team meeting is held to discuss the death; then a meeting was held at the woman's local health facility or district hospital, depending on where the death occurred, and included health center staff, district hospital staff, and community health workers, with participants agreeing on a cause of death; then a public meeting in the woman's local community, attended by health representatives and local community members presented on the death and action points to prevent future deaths; bi-monthly meeting for community and health facility representatives to hear about the system; quarterly traditional leadership meeting to share innovations and lessons learned^4^ - Two community health workers assigned to each group of 1,000 people in a camp and given a weekly report form to record births and deaths; community health workers conducted verbal autopsies; weekly supervision meetings and deaths were collated by supervisor and entered into system; cross-checked with clinical records; weekly graveyard counts conducted by a graveyard watcher; community health workers confirmed graveyard watch deaths with community leaders^5^ - Village health workers identified and registered all pregnant women within their assigned area; interviewed pregnant women during birth planning and educational visits; displayed maternal death data on a community board (generally during their fifth month of pregnancy)^6^ - Health extension workers visited homes within hours or days after a pregnancy ended and assessed and registered birth and birth conditions; health extension workers continued follow-up until a maternal death occurred or the individual reached 42 days postpartum; husbands and fathers of the baby were primary sources of information, or other family members if needed; registered deaths in birth registry books; sent a copy of registration to hospital; final home visit six weeks after birth or abortion when death information was not obtained prior^7^ | **2** |
| *Definitions* | NA | - Maternal death as death of a pregnant woman or within 42 days of termination of pregnancy from any cause related to the pregnancy; if no trauma is present and the pregnancy terminates at less than 20 weeks, the cause of death is considered to be abortion-related^1^ - Maternal death as death of a pregnant woman or within 42 days of termination of pregnancy from any cause related to the pregnancy^4,7^ - Deaths within 42 days of a pregnancy outcome^2^ - Deaths to individuals 12-49 years old; maternal deaths only using ICD-10^3^ - Only included deaths related to singletons^6^ - Deaths during pregnancy and at least through 42 days postpartum^6^ | **2** |
| *Sample size* | NA | - 156,309 pregnant women screened, 158,205 enrolled; 155,952 had data with delivery outcomes; 221 maternal deaths^1^ - Population of 228,186, with 13,602 births, and 323 deaths^2^ - Population of 22,000 people; 856 births and 164 deaths^3^ - Population of 456,500 people; 52 deaths^4^ - 904 pregnant individuals and 4 maternal deaths^6^ - Population of 488,287 people; 53 maternal deaths among registered births^7^ | **4** |
| *Timing of point estimate relative to data collection* | NA | - Prospective study for 12 months^3^ - Prospective study for 12 months^4^ - Deaths reported monthly and four-monthly, but unclear for which time periods^5^ - Only hours or days after the death did health extension workers visit homes to collect and send in data^7^ | **4** |
| *Bias* | NA | - Requires consent of pregnant individual to be entered into the registry^1^ - Uses an algorithm to assign cause of death, though this is compared to provider assigned cause of death^1^ - Interview two relatives to determine cause of death; if informant conflicted, interviewed a third individual^3^ - Difficulty reaching transient communities^4^ - Using one data form allowed possibility of loss of data^4^ - Miss deaths that occur before five months, due to registration "generally during the fifth month of pregnancy"^6^ - Interviewed the assumed father of the decedent, who may not have known about abortion intentions or action^7^ - Validated data by conducting a house-to-house survey in 15 of the 30 included villages^7^ | **2.5** |
|  |  |  |  |
| *Human resources* | NA | - Nurses/healthcare workers to collect data for the registry and to assign cause of death^1^ - One key informant per 250 households (traditional birth attendants); an interviewer to verify information; one full time salaried interviewer for each cluster (12 clusters); supervisor interviews provider and family member; two obstetricians assign cause of death^2^ - Seven mid-level providers (one health officer and six nurse-midwives) and twenty lay healthcare workers (traditional birth attendants and community-based reproductive health agents) received three weeks of intensive training in sentinel surveillance, vital registration, and verbal autopsy^3^ - Traditional birth attendants, community-based reproductive health agents, and local priests received five days of training on the study protocol and sentinel surveillance^3^ - Health extension workers received five days of intensive training, including comprehensive background information and training in the study protocol, sentinel surveillance methodology, data collection and management for civil vital registration^3^ - Nurses and nurse-midwives received seven days of training on study protocol, in-depth interview techniques, verbal autopsy techniques, and techniques for addressing sensitive subjects and another four days on cause of death attribution^3^ - Meetings with all research team members monthly for the first three months of implementation^3^ - Trained 350 community teams, made up of group village headman, community health workers, and volunteers; one team for each health center providing maternity care^4^ - Two to three village health workers trained in each village on implementing the surveillance system and general reproductive health^6^ | **2** |
| *Time needed for implementation* | NA | - Not reported, but mention verbal autopsy occurred 6-8 weeks after death^2^ - At least one month of training, then prospective, and timing not reported^3^ - Identified in discussion as a "lengthy process"^4^ | **3** |
| *Data collection training* | NA | - Trained on collecting data from participants as well as how to assign cause of death^1^ - "Detailed training" for priests, traditional birth attendants, and community-based reproductive health agents on how to educate and motivate families to report births and deaths in their home^3^ - One week training at each woreda office with health extension workers, supervisors, and district health authorities; supervisors were trained nurses who helped the HEWs learn about reviewing deaths^4^ | **2** |
| *Statistical training* | NA | - Used a computer system to assign cause of death^1^ - Used WHO methodology to assign cause of death^3^ | **4** |
| *Digitalization* | NA | - Not a problem to digitalize^1–7^ | **4** |
| *Cost* | NA | - Informants paid US$0.65 for every accurate birth or death identification; monthly cost of US$2,759.00, including management was US$3,103.00; training and recruitment of staff cost US$7,355.00^2^ - $0.90 per resident of the catchment area: ~US$19,800.00^3^ | **3** |
| *Total score* | | | **32.5/44** |

**References**

1. Pasha O, McClure EM, Saleem S, et al. A prospective cause of death classification system for maternal deaths in low and middle-income countries: results from the Global Network Maternal Newborn Health Registry. *BJOG*. 2018;125(9):1137-1143. doi:10.1111/1471-0528.15011

2. Barnett S, Nair N, Tripathy P, Borghi J, Rath S, Costello A. A prospective key informant surveillance system to measure maternal mortality - findings from indigenous populations in Jharkhand and Orissa, India. *BMC Pregnancy Childbirth*. 2008;8:6. doi:10.1186/1471-2393-8-6

3. Prata N, Gerdts C, Gessessew A. An innovative approach to measuring maternal mortality at the community level in low-resource settings using mid-level providers: a feasibility study in Tigray, Ethiopia. *Reprod Health Matters*. 2012;20(39):196-204. doi:10.1016/S0968-8080(12)39606-7

4. Bayley O, Chapota H, Kainja E, et al. Community-linked maternal death review (CLMDR) to measure and prevent maternal mortality: a pilot study in rural Malawi. *BMJ open*. 2015;5(4):e007753.

5. Bowden S, Braker K, Checchi F, Wong S. Implementation and utilisation of community-based mortality surveillance: A case study from Chad. *Conflict and health*. 2012;6:11. doi:10.1186/1752-1505-6-11

6. Kilonzo A, Kouletio M, Whitehead SJ, Curtis KM, McCarthy BJ. Improving surveillance for maternal and perinatal health in 2 districts of rural Tanzania. *Am J Public Health*. 2001;91(10):1636-1640. doi:10.2105/ajph.91.10.1636

7. Yaya Y, Data T, Lindtjørn B. Maternal mortality in rural south Ethiopia: outcomes of community-based birth registration by health extension workers. *PLoS One*. 2015;10(3):e0119321. doi:10.1371/journal.pone.0119321
