# Supplementary material for: Maternal mortality estimation methodologies: a scoping review and evaluation of suitability for use in humanitarian settings
Source: Confl Health. 2024 Dec 19;18:75. doi: 10.1186/s13031-024-00636-y (PMC11657123; doi:10.1186/s13031-024-00636-y)
Supplement: Supplementary file 5 — Additional file 5. Neighborhood methodology completed evaluation form. Additional file 5 shows the completed evaluation form for the neighborhood methodology. [file 13031_2024_636_MOESM5_ESM.docx]

**Additional file 5. Neighborhood methodology completed evaluation form**

| **Category** | **The neighborhood method (Alam, et al, 2014)**^1^ | | |
| --- | --- | --- | --- |
|  | **Notes from original implementation** | **Notes from additional implementations** | **Score (1-4)** |
| *Summary of methodology* | Adult women were asked about any maternal deaths; multiple births; infant deaths, live births, and some other events they knew of in a small, specified area around their home. | | |
| *Data sources* | Any woman present in the bari (neighborhood) | Not only women — anyone present in key gathering points in the village (such as markets and tea stalls invited to participate); drew a map of the village with participant assistance and asked for names of those with deaths and key informants; visited homes of decedents to confirm cause of death and to ask about other deaths; also key informant interviews^2^ | **2** |
| *Definitions* | Deaths during pregnancy, delivery, and within 42 days of delivery | ICD-10^2^ | **3** |
| *Sample size* | No specific sample size, based on number of deaths recalled | NA | **3** |
| *Timing of point estimate relative to data collection* | Adds three years and 42 days (for data collection) | NA | **1** |
| *Bias* | - Selection bias (only individuals known to have been pregnant by community members) - Non-response bias (at home verbal autopsies) - Selection bias (miss early deaths [i.e., before someone knows they are pregnant, and the community knows]) - Selection bias (assumes that everyone has a family member that can be interviewed for verbal autopsy, who knows it was a maternal death and the cause of death) - Selection bias (map of area may not be accurate and individuals may not report on their entire neighborhood) - Recall bias (three-year window of reporting) - Selection bias (self-appointed spokeswoman for each group that had to be a married woman 18+ years, who may change aggregated submission based on responses of peers or based on own recollection | NA | **2** |
|  |  |  |  |
| *Human resources* | Not reported | Two field teams; four data collectors and one field supervisor (CKA) ^2^ | **2.5** |
| *Time needed for implementation* | 42 days of data collection | 84 person-hours of data collection for five unions (geography) and 82 verbal autopsies^2^ | **2.5** |
| *Data collection training* | 1/2 day of training | NA | **3** |
| *Statistical training* | Simple calculation | "As a check on data completeness, the team determined whether the number of deaths identified using the community knowledge approach was at least 80% of the probable number of deaths in the village in the previous 3 years, calculated by multiplying the village population estimate from the 2011 population census by the crude death rate in rural Bangladesh of six per 1,000 persons per year.24,28 If the number of deaths was fewer than 80% of the probable number of deaths, the team checked the list with another group of village residents, and repeated the aforementioned procedure."^2^ | **4** |
| *Digitalization* | Easy to digitize |  | **4** |
| *Cost* | Not reported | NA | **3** |
| *Total score* | | | **29/44** |

**References**

1. Alam N, Townend J. The neighbourhood method for measuring differences in maternal mortality, infant mortality and other rare demographic events. *PLoS One*. 2014;9(1):e83590. doi:10.1371/journal.pone.0083590

2. Paul RC, Gidding HF, Nazneen A, et al. A Low-Cost, Community Knowledge Approach to Estimate Maternal and Jaundice-Associated Mortality in Rural Bangladesh. *Am J Trop Med Hyg*. 2018;99(6):1633-1638. doi:10.4269/ajtmh.17-0974
