# Supplementary material for: Maternal mortality estimation methodologies: a scoping review and evaluation of suitability for use in humanitarian settings
Source: Confl Health. 2024 Dec 19;18:75. doi: 10.1186/s13031-024-00636-y (PMC11657123; doi:10.1186/s13031-024-00636-y)
Supplement: Supplementary file 6 — Additional file 6. Motherhood methodology completed evaluation form. Additional file 6 shows the completed evaluation form for the motherhood methodology. [file 13031_2024_636_MOESM6_ESM.docx]

**Additional file 6. Motherhood methodology completed evaluation form**

| **Category** | **The motherhood method (Maskey, et al, 2011)**^1^ | | |
| --- | --- | --- | --- |
|  | **Notes from original implementation** | **Notes from additional implementations** | **Score (1-4)** |
| *Summary of methodology* | 7-15 pregnant women per village or ward met once a month to discuss issues related to mother and child health; female community health volunteers facilitated the meetings; births, maternal deaths, infant death collected from village development committees over a two year period; Female community health volunteers collected list of mothers who gave birth by collecting BCG and TT vaccination information from registries; Female community health volunteers looked at pregnancy outcomes of the individuals on the list; mothers on the list were asked about date of birth or baby/babies; also collected information on maternal deaths, infant deaths, stillbirths, abortions, conflict in group opinion resolved with interview with woman in question or another household member; for those that died during the period, a close relative was interviewed; validated results with a census of remaining households not in the list of study births | | |
| *Data sources* | - Vaccination records and mothers' input group (or relative if there is disagreement about cause of death) - 7-15 pregnant women living in the same village or wards who meet once a month to discuss issues related to maternal and child health | NA | **2** |
| *Definitions* | No clear definition, just "maternal deaths" | NA | **1** |
| *Sample size* | No specific sample size, based on number of deaths recalled | NA | **3** |
| *Timing of point estimate relative to data collection* | Adds two years and three months; estimate for prior two years + plus three months before study | NA | **1** |
| *Bias* | - Selection bias (misses those without vaccines) - Selection bias (who community leaders know about, miss people not registered, miss early deaths) - Non-response bias (at home verbal autopsies) - Selection bias (miss early deaths [i.e., before someone knows they are pregnant, and the community knows]) - Selection bias (assumes that everyone has a family member that can be interviewed for verbal autopsy, who knows that it was a maternal death and who knows cause of death) - Need to then validate against consensus not in list of study births - 25% undercount compared to census for live births and total births | NA | **2** |
|  |  |  |  |
| *Human resources* | Two-day training for supervisors and one female health volunteer per mothers' group | NA | **3** |
| *Time needed for implementation* | Two years of data on vaccination, three-month lag, one meeting 5 days per village | NA | **2.5** |
| *Data collection training* | Pregnant women's group of 10-15 months and local FHVs: review pregnancy outcomes of individuals with TT or BCG vaccine | NA | **4** |
| *Statistical training* | Simple calculation | NA | **4** |
| *Digitalization* | Easy to digitize | NA | **4** |
| *Cost* | US$10,986.00 to identify 49 deaths among 15,161 births (i.e., US$4.40 per death) | NA | **3** |
| *Total score* | | | **29.5/44** |

**References**

1. Maskey MK, Baral KP, Shah R, Shrestha BD, Lang J, Rothman KJ. Field test results of the motherhood method to measure maternal mortality. *Indian J Med Res*. 2011;133(1):64-69.
