# Supplementary material for: Maternal mortality estimation methodologies: a scoping review and evaluation of suitability for use in humanitarian settings
Source: Confl Health. 2024 Dec 19;18:75. doi: 10.1186/s13031-024-00636-y (PMC11657123; doi:10.1186/s13031-024-00636-y)
Supplement: Supplementary file 7 — Additional file 7. Indirect sisterhood methodology completed evaluation form. Additional file 7 shows the completed evaluation form for the indirect sisterhood methodology. [file 13031_2024_636_MOESM7_ESM.docx]

**Additional file 7. Indirect sisterhood methodology completed evaluation form**

| **Category** |  | **The indirect sisterhood method (Graham, et al, 1989)**^1^ |  |
| --- | --- | --- | --- |
|  | **Notes from original implementation** | **Notes from additional implementations** | **Score (1-4)** |
| *Summary of methodology* | Interview a random sample of all adults over age 15 years in target location (Graham, et al., used a surveillance system's list). Instructed to question the named individuals directly and to make three calls before resorting to a proxy. Asked four questions: How many sisters (born to the same mother) have you ever had who were ever-married (including those who are now dead)? How many of these ever-married sisters are alive now? How many of these ever-married sisters are dead? How many of these dead sisters died while they were pregnant, or during childbirth, or during the six weeks after the end of pregnancy? Graham, et al. report asking about ever-marriage to avoid asking about age (in case of recall bias). | | |
| *Data sources* | - Requires accurate census data; Verbal autopsy + census data = two sources - Asks any individual 15+ years old about any married sister | Survey respondents:   - Any sibling(s) 15+ years old; unclear if all siblings in a household are interviewed^2–6^ - Any sibling(s) 15-49 years old; unclear if all siblings in a household are interviewed^7–9^ - Any sibling(s) 15-50 years old; unclear if all siblings in a household are interviewed^10^ - All siblings 15+ years old present in the household at time of interview^11^ - One sibling 12-49 years old^12^ - One sibling 12+ years old^13^ - One sibling per household selected via lottery^14^ - Any female sibling(s) 15-49 years old; unclear if all female siblings in a household are interviewed ^15–21^ - One female sibling 15-49 years old^22,23^ - One female sibling 15-49 years old who was the wife of the household head or female household head; if more than one eligible, one respondent was randomly selected^24^ - Any female sibling(s) 15+ years old; unclear if all female siblings in a household are interviewed^25^ - Any female sibling(s) attending antenatal care in the area aged 15-59 years old, any male/female siblings aged 15-60 years old from a household survey of all households, or respondents by proxy; only one eligible sibling per family^26^ - All women present in the household 18+ years old and not sisters^27^ - All individuals 15+ years old living in a household not born of the same mother and present at the time of study are interviewed^28^ - All household members 15-60 years old^29^ - All household members 15+ years old if there was a pregnancy in the last five years^30^   Decedents:   - Any female sibling 10+ years old^21^ - Any female sibling 13-50 years old^31^ - Any female sibling 14-44 years old^32,33^ - Any female sibling 15+ years old^3,4,6–8,12,14,16–20,23–27,30,34^ - Any ever-married female sibling^10,11,15,22^ - Any ever-married or 15+ years old female sibling^9^ - Any ever-married 15-49 year old female sibling^5^ - Any female sibling 15-49 years old^2^ - Any female sibling^28,29^ - No age or other restrictions, age of sexual activity derived from life tables^13^ | **2** |
| *Definitions* | Report use of ICD-9 definition of maternal mortality (within 42 days of birth), but are actually using pregnancy-related (within one year) | - No definition provided^10^ - Pregnancy and childbirth^28^ - ICD-9, ICD-10, or something consistent with those classifications^2–9,11–27,29–31,34,35^ - "Maternity-related deaths" and those within 90 days of delivery or termination^32,33^ | **3** |
| *Sample size* | 3,000-6,000 adults (aka. interviews) required for a "broad" estimate | - In order to estimate the MMR with 80% power and 95% confidence level, given a mortality level between 1,500 and 2,500 per 100,000 live births, at least 3000 sisters are needed.^31^ - Using this formula, the calculated sample size for this study was around 2,100 households. To account for cluster sampling in the Ibadan North LGA, 3,000 respondents were required for the study. Therefore 3,000 households were selected from the political wards of Ibadan North and Ido Local Government Area of Oyo state.^7,8^ - The sample size of respondents was calculated based on the assumption of the proportion of respondents to maternal death questionnaire to be 50%, and a tolerable error of 5%. This was to increase the sample size and precision as a previous 19 study used 45%. In order to allow for the cluster sampling design effect, the value was rounded to the next 1,000. The figure was increased by 10% for possible none or poor responses.^9^ - We assumed a maternal mortality rate of 820/100,000 as indicated by WHO/UNICEF in 1997 and assumed this rate to be only 50% accurate (410/100,000 to 1230/100,000) and used an estimate of 300/100,000, which is the lower limit of the confidence intervals for this 1997 estimate. The number of respondents (according to the indirect Sisterhood Method) needed to establish a maternal mortality rate of 300 maternal deaths/100 000 live births per year within 20% was 4000 household surveys. To account for cluster sampling in Herat City, we planned to include 5000 households in the study. A total of 5014 households were selected from 7 districts in 34 established (non-nomadic) villages.^15^ - Using a TFR of 6.0, an estimated MMR of 230/100 000 livebirths, and a tolerated error of 15%, the estimated sample size for the health facility-based study was 8500. A target sample size of 10 000 was set to allow for incomplete interviews and other problems.^16^ - The sample size required was first calculated on the basis of numbers of women needed to provide a recent estimate of childhood mortality. The sample size needed to obtain the single best estimate of maternal mortality using the sisterhood method proved to be well within the quota of 100 ever-married women per cluster. Assuming a maternal mortality ratio of about 500 per 100 000 live births, a total fertility rate of about 6, and a tolerated error of 15%, almost 4000 adult respondents were needed.^17^ - The sample size calculation was based on the following premises: (1) The reference sizes established by the authors of the method.14 Samples of 3,000 to 6,000 adults are recommended while other authors define as acceptable a sample size of 2500 to 3000 respondents.15 (2) The number of deaths needed to estimate the MMR, with a specific margin of error is calculated from Equation 1, r > zα/22 * (100/% margin of error)2, (where r = number of deaths needed to estimate the MMR and % margin of error = percentage margin of error) with 95% confidence interval and a 20% margin of error, the equation leads to a required number of at least 97 deaths. Empirically, from other published studies in similar settings, to detect 97 deaths with an estimated MMR of about 500 per 100 000 live births, the sample size needed is 3200 respondents.^20^ - According to the method of Hanley et al," in case the expected MMR is between 500 and 750, between 2,100 and 3,200 interviews are needed. Total number of maternal deaths margin of <20% error. To achieve a margin of +10% error one would need 8,000-13,000 interviews. With TFR, LTR may be translated into MMR using large numbers were not feasible in our setting the following formula: and, therefore, we chose to interview 3,000 people, aiming at a margin of 20% error^34^ - In settings with high levels of maternal mortality (over 500 maternal deaths per 100,000 live-births), the sample size recommended for the sisterhood method is in the order of 4,000 households or less (Hanley et al 1996). The sample size used for this study was 4,315 households. Therefore, the sample size used in this study was sufficient, as it is large enough to detect an MMR of 250–500 with an error margin of 20% and a 95% confidence level. Sampling units were selected by multistage sampling.^5^ | **2** |
| *Timing of point estimate relative to data collection* | Calculates lifetime risk for five-year age groups | 5-year recall of births and deaths^14^ | **1** |
| *Bias* | - Selection bias (only asks about ever-married sisters) - Non-response bias (at home interviews) - Recall bias (could name any death) - Selection bias (miss early deaths [i.e., before others know about pregnancy]) - Selection bias (assumes that someone tells siblings about pregnancy, that they know it was a maternal death, and that they know the cause of death) - Selection bias (double counting if an individual has several siblings) - Selection bias (assumes that the individual that died has sisters AND that they were present at death) | NA | **2** |
| *Human resources* | - Trained eight fieldworkers, conducted 2,163 interviews over five days with six workers a day | - The survey interviews were conducted by 48 Afghan women who were trained and supervised by the PHR field supervisor and 4 trained Afghan research team leaders^15^ - Implemented by 10 nurse-midwife students^2^ - Twenty male and 17 female interviewers, all nursing students, were available for the survey. The suitability of using men to ask questions of women in a predominantly Muslim area has to be decided in each local set- ting. In addition, there were six female supervisors. Each of the 17 interviewing teams comprised three interviewers and one supervisor. Data collected over 10 days^17^ - Eight field assistants (four men and four women) participated in the household survey, and five field assistants (all women) were trained for the work in the antenatal clinics. Six of the field assistants had some type of education beyond standard seven, while seven had standard seven. They were trained in interviewing techniques over a period of several weeks.^26^ - 26 students of clinical officers training center were trained for two days^34^ - A total of 20 interviewers and five supervisors were trained to collect data. Door-to-door interviews were conducted among all residents in the selected EAs that met the inclusion criteria for the study until the desired sample size^5^ - 6 hours/day for seven days^29^ | **2** |
| *Time needed for implementation* | - Five days to collect data with six staff | - Seven days of data collection (unknown number of research assistants)^9^ - All interviews were conducted over a 10-day period in March 2002. Interviews with participants lasted approximately 20 to 30 minutes and were conducted in a private setting with no one else present.^15^ - Data were collected in June and July of 1992.^16^ - The fieldwork for the survey took place between July and August 2011^18^ - During the first update round (January to June 2011) from 17,173 WRA^19^ - 4179 women interviewed from February 1995 to March 1996 at first antenatal care visit^26^ - Two months of data collection^3^ - Seven months to cover 24 villages^10^ - Two-week data collection period^34^ - Two months (8 weeks) of data collection with 23 student nurses^4^ - 52 interviewers and 26 supervisors completed in 14 days: 42 respondents/14 households) daily per interviewer^29^ - 10 research assistants over 12 days^23^ - Two months with secondary school student interviewers female^6^ | **2** |
| *Data collection training* | - One hour plus an afternoon of supervision | - 5 days of intensive training with 10 staff^7,8^ - 2 days of training with research assistants^9^ - Researcher training consisted of 3 days of classroom teaching and role-play, followed by several days of field observation and continuous supervision.^15^ - Health personnel, mostly nurses, at each facility were taught to administer the sisterhood method questionnaire during a one-day training session. Most of the interviewers were nurses, but a few were physicians or health educators.^16^ - During the five-day training of the interviewers, one day was devoted to the translation of the questionnaire. Interviewers were divided into three groups according to their mother tongue. Each group had a supervisor who ensured that each question was dis- cussed and translated into Afar, Arabic or Somali, with consensus within the group on the choice of words and syntax.^17^ - One day of training on sisterhood as part of a larger training on survey deployment^14^ - 15 nursing students were trained for two half-days, which included field testing in the villages surrounding the nursing school area & in other district 80 primary school teachers were trained in a similar way, including a small field test, in 11 groups^3^ - One month of training^13^ - Trained for two days^30^ | **3** |
| *Statistical training* | - Simple calculation, but requires accurate census data | - Additional adjustment: A correction made for the fact that respondents under the age of 25 are likely to have more sisters who will reach age 15 or get married in the future. Therefore, the number of respondents in the age group under the age of 25 was multiplied by the average number of sisters of those in the age group above 25. This average was 2.29 (3,410/1,491), that is each respondent above the age of 25 had 2.29 sisters above 15 years of age.^2^ - The survey data give a current TFR of between 6 and 7 births per woman. The sex ratio for children ever born was 1.15, indicating under-counting of female births. Two estimates of fertility could thus be obtained from these data by adjusting for this under-counting. The first (unadjusted) estimate of the TFR was 5.5 and the latter (adjusted) estimated the TFR to be 6.8 live births per woman.^17^ | **3** |
| *Digitalization* | - Easy to digitize | NA | **4** |
| *Cost* | - Cost not reported | NA | **2** |
| *Total score* | | | **26/44** |

**References**

1. Graham W, Brass W, Snow RW. Estimating Maternal Mortality: The Sisterhood Method. *Studies in Family Planning*. 1989;20(3):125-135. doi:10.2307/1966567

2. Beltman JJ, van den Akker T, Lam E, et al. Repetition of a sisterhood survey at district level in Malawi: the challenge to achieve MDG 5. *BMJ Open*. 2011;1(1):e000080. doi:10.1136/bmjopen-2011-000080

3. Le Bacq F, Rietsema A. High maternal mortality levels and additional risk from poor accessibility in two districts of northern province, Zambia. *Int J Epidemiol*. 1997;26(2):357-363. doi:10.1093/ije/26.2.357

4. Oosterhuis JW. Estimating maternal mortality by sisterhood method in rural Zimbabwe. *Trop Doct*. 1993;23(2):67-68. doi:10.1177/004947559302300210

5. Oye-Adeniran BA, Odeyemi KA, Gbadegesin A, et al. The use of the sisterhood method for estimating maternal mortality ratio in Lagos state, Nigeria. *J Obstet Gynaecol*. 2011;31(4):315-319. doi:10.3109/01443615.2011.561381

6. Vork FC, Kyanamina S, van Roosmalen J. Maternal mortality in rural Zambia. *Acta Obstet Gynecol Scand*. 1997;76(7):646-650. doi:10.3109/00016349709024604

7. Adegoke AA, Campbell M, Ogundeji MO, Lawoyin TO, Thomson AM. Community Study of maternal mortality in South West Nigeria: how applicable is the sisterhood method. *Matern Child Health J*. 2013;17(2):319-329. doi:10.1007/s10995-012-0977-z

8. Adegoke AA, Campbell M, Ogundeji MO, Lawoyin T, Thomson AM. Place of birth or place of death: an evaluation of 1139 maternal deaths in Nigeria. *Midwifery*. 2013;29(11):e115-121. doi:10.1016/j.midw.2012.11.018

9. Aminat ZY, Aliyu A, Tukur D. Estimation of maternal mortality using the indirect sisterhood method in suleja, Niger state-Nigeria. *Journal of Medicine and Biomedical Research*. 2013;12:131-138.

10. Mace R, Sear R. Maternal mortality in a Kenyan pastoralist population. *Int J Gynaecol Obstet*. 1996;54(2):137-141. doi:10.1016/0020-7292(96)02691-4

11. Soemantri S. Population Based Estimates of Maternal Mortality in Mojokerto, East Java (the Application of Indirect Technique: Sisterhood Method). *Indonesian Bulletin of Health Research*. 1989;17(4):20516.

12. Hernández B, Chirinos J, Romero M, Langer A. Estimating maternal mortality in rural areas of Mexico: the application of an indirect demographic method. *Int J Gynaecol Obstet*. 1994;46(3):285-289. doi:10.1016/0020-7292(94)90407-3

13. Lech MM, Zwane A. Survey on maternal mortality in Swaziland using the sisterhood method. *Paediatr Perinat Epidemiol*. 2002;16(2):101-107. doi:10.1046/j.1365-3016.2002.00411.x

14. Kea A, Lindtjorn B, Tekele A, Hinderaker S. Reduction in maternal mortality ratio varies by district in Sidama Regional State, southern Ethiopia: Estimates by cross-sectional studies using the sisterhood method and a household survey of pregnancy and birth outcomes. Published online October 4, 2022. doi:10.1101/2022.10.02.22280613

15. Amowitz L, Reis C, Iacopino V. Maternal Mortality in Herat Province, Afghanistan, in 2002: An Indicator of Women’s Human Rights. *JAMA : the journal of the American Medical Association*. 2002;288:1284-1291. doi:10.1001/jama.288.10.1284

16. Danel I, Graham W, Stupp P, Castillo P. Applying the sisterhood method for estimating maternal mortality to a health facility-based sample: a comparison with results from a household-based sample. *Int J Epidemiol*. 1996;25(5):1017-1022. doi:10.1093/ije/25.5.1017

17. David P, Kawar S, Graham W. Estimating maternal mortality in Djibouti: an application of the sisterhood method. *Int J Epidemiol*. 1991;20(2):551-557. doi:10.1093/ije/20.2.551

18. Doctor H, Findley S, Afenyadu G. Estimating Maternal Mortality Level in Rural Northern Nigeria by the Sisterhood Method. *International Journal of Population Research*. 2012;464657. doi:10.1155/2012/464657

19. Doctor HV, Olatunji A, Findley SE, Afenyadu GY, Abdulwahab A, Jumare A. Maternal mortality in northern Nigeria: findings of a health and demographic surveillance system in Zamfara State, Nigeria. *Trop Doct*. 2012;42(3):140-143. doi:10.1258/td.2012.120062

20. Font F, Alonso González M, Nathan R, et al. Maternal mortality in a rural district of southeastern Tanzania: an application of the sisterhood method. *Int J Epidemiol*. 2000;29(1):107-112. doi:10.1093/ije/29.1.107

21. Ngom P, Akweongo P, Adongo P, Bawah AA, Binka F. Maternal mortality among the Kassena-Nankana of northern Ghana. *Stud Fam Plann*. 1999;30(2):142-147. doi:10.1111/j.1728-4465.1999.00142.x

22. Gulumbe U, Alabi O, Omisakin OA, Omoleke S. Maternal mortality ratio in selected rural communities in Kebbi State, Northwest Nigeria. *BMC Pregnancy Childbirth*. 2018;18(1):503. doi:10.1186/s12884-018-2125-2

23. Usman NO, Abdullahi HM, Nmadu AG, Omole VN, Ango JT. Estimation of maternal mortality by sisterhood method in two rural communities in Kaduna State, Nigeria. *Journal of Medicine in the Tropics*. 2019;21(2):62.

24. Sharma V, Brown W, Kainuwa MA, Leight J, Nyqvist MB. High maternal mortality in Jigawa State, Northern Nigeria estimated using the sisterhood method. *BMC Pregnancy Childbirth*. 2017;17(1):163. doi:10.1186/s12884-017-1341-5

25. Garenne M, Sauerborn R, Nougtara A, Borchert M, Benzler J, Diesfeld J. Direct and indirect estimates of maternal mortality in rural Burkina Faso. *Stud Fam Plann*. 1997;28(1):54-61.

26. Evjen-Olsen B, Hinderaker S, Kazaura M, Bergsjø P, Gasheka P, Kvåle G. Estimates of maternal mortality by the sisterhood method in rural northern Tanzania: A household sample and an antenatal clinic sample. *BJOG : an international journal of obstetrics and gynaecology*. 2000;107:1290-1297.

27. Liese KL, Pauls H, Robinson S, Patil C. Estimating Maternal Mortality in Remote Rural Regions: an Application of the Sisterhood Method in Tajikistan. *Cent Asian J Glob Health*. 2019;8(1):341. doi:10.5195/cajgh.2019.341

28. Orach CG. Maternal mortality estimated using the Sisterhood method in Gulu district, Uganda. *Trop Doct*. 2000;30(2):72-74. doi:10.1177/004947550003000205

29. Wirawan DN, Linnan M. The Bali Indirect Maternal Mortality Study. *Stud Fam Plann*. 1994;25(5):304-309.

30. Yaya Y, Lindtjørn B. High maternal mortality in rural south-west Ethiopia: estimate by using the sisterhood method. *BMC Pregnancy Childbirth*. 2012;12:136. doi:10.1186/1471-2393-12-136

31. Aa I, Grove MA, Haugsjå AH, Hinderaker SG. High maternal mortality estimated by the sisterhood method in a rural area of Mali. *BMC Pregnancy Childbirth*. 2011;11:56. doi:10.1186/1471-2393-11-56

32. Shahidullah M. The sisterhood method of estimating maternal mortality: the Matlab experience. *Stud Fam Plann*. 1995;26(2):101-106.

33. Shahidullah M. A comparison of sisterhood information on causes of maternal death with the registration causes of maternal death in Matlab, Bangladesh. *Int J Epidemiol*. 1995;24(5):937-942. doi:10.1093/ije/24.5.937

34. Mbaruku G, Vork F, Vyagusa D, Mwakipiti R, van Roosmalen J. Estimates of maternal mortality in western Tanzania by the sisterhood method. *Afr J Reprod Health*. 2003;7(3):84-91.

35. Walraven GE, Mkanje RJ, van Roosmalen J, van Dongen PW, Dolmans WM. Assessment of maternal mortality in Tanzania. *Br J Obstet Gynaecol*. 1994;101(5):414-417. doi:10.1111/j.1471-0528.1994.tb11914.x
