# Supplementary material for: Maternal mortality estimation methodologies: a scoping review and evaluation of suitability for use in humanitarian settings
Source: Confl Health. 2024 Dec 19;18:75. doi: 10.1186/s13031-024-00636-y (PMC11657123; doi:10.1186/s13031-024-00636-y)
Supplement: Supplementary file 8 — Additional file 8. Reproductive Age Mortality Study (RAMOS) methodology completed evaluation form. Additional file 8 shows the completed evaluation form for the Reproductive Age Mortality Study (RAMOS) methodology. [file 13031_2024_636_MOESM8_ESM.docx]

**Additional file 8. Reproductive Age Mortality Study (RAMOS) methodology completed evaluation form**

| **Category** | **The Reproductive Age Mortality Study (RAMOS) method (Fortney & Grubb & et al., 1986/1987/1988)**^1–4^ | | |
| --- | --- | --- | --- |
|  | **Notes from original implementation** | **Notes from additional implementations** | **Score (1-4)** |
| *Summary of methodology* | In Egypt, vital registration records provide information on deaths to women of reproductive age (15-49) and in Indonesia, these data are collected by community health workers in coordination with local village leaders. Trained interviewers go out and interview surviving members of the household about the death. | | |
| *Data sources* | Used vital records plus interviews in Egypt; used community health workers' monthly visits to village heads plus interviews in Indonesia; triangulation of data | - Used demographic surveillance site data, community health worker interviews, verbal autopsy, and physician review^5^ - Survey of 10,135 households including deaths of household members in the five years preceding the survey, verbal autopsy (by female doctors) for WRA, three leading obstetricians independently reviewed the interview and assigned cause of death, the three obstetricians met about any disagreements^6,7^ - Population-based surveys specifically on maternal and infant mortality and associated risk factors to all households which obtained information on deaths, second questionnaire administered to identified ever-married women 15-54 who were current residents of the sampled households about reproductive health; also sought information about deaths from other key informants (village leaders, preachers of local mosque, traditional birth attendants, staff of health facilities); second round of interviews, reported deaths of WRA investigated through verbal autopsy 4-12 weeks after first found by lady health visitors or female medical technicians, then reviewed by independently by senior obstetricians and family physicians who determined cause of death and disagreements resolved by consensus^8^ - Reviewed deaths reported in a district over 3.5 years, conducted verbal autopsy for all deaths not in the RAMOS year (2008) and that did not have a verbal autopsy already; met with the family of the deceased individual, traditional birthing attendant, or local health worker; questionnaire was administered in a home or clinic^9^ - Two house listers visited the main village and every major hamlet of all villages and identified key informants (auxiliary nurse midwives, child nutrition workers, local self-government members, shopkeepers, and 2-3 elderly women); enquired about all women 12-60 years who had died over the past year and narrowed down deaths to WRA; lists were validated by two research investigators and one research supervisor who visited each village and contacted the decedents families and verified the date or death and age, checked about other deaths in the village in the study period and revised the list (made up to three visits in order to meet the most reliable respondent about the death); then returned for the verbal autopsy; one doctor from the research group assigned the cause of death; form then sent to one external reviewer from a panel of reviewers and the cause assigned by the first doctor was blinded; for pregnancy-related deaths, the reviewer was a gynecologist and for other deaths there was one surgeon and two physicians; for disagreements, resolved in conversation^10^ - Collect data annually by a team of 10 local fieldworkers, specially trained fieldworkers conducted verbal autopsies in each household where a death had been recorded with the person most closely associated with the decedent; clinician assessment was used to determine verbal autopsy diagnoses; completed questionnaires were reviewed independently by two physicians; if they did not agree, a third practitioner was included; if no agreement (2/3), the cause of death was "undetermined"^11–13^ - Deaths identified by clerks at local health units and health registration bureaus and by specially trained social workers; health bureau clerks reported each death every month; social workers interviewed family members of all WRA (15-50 years)^2^ - In each cluster, death events were monitored and registered by appointed notification assistants from the locality; then reported on s special form; within two weeks, the research assistant visited the bereaved family and conducted a verbal autopsy; if needed, hospitals and physicians were contacted to finish filling out the form; forwarded to the central office of the study where it was reviewed by two physicians; if necessary, forms were returned to the research assistant or field for further clarification^14^ - Deaths identified after half-day awareness meetings at health facility (district health management team, health facility in-charges, healthcare providers, zone coordinators, cluster supervisors) and community level (senior health surveillance assistants, traditional authorities, groups village headmen, individual village headman); meetings conducted over a period of three weeks; at facility level, deaths of WRA identified by providers and notified to research staff, mortuary registers reviewed, at community level, deaths of WRA identified via heads of households, village leaders, traditional healers, burial sites, village registers, traditional birth attendants, and police stations; upon receiving a report of any death, tr4ained staff obtained more information using a verbal autopsy; visited all health facilities once a month, cross-checked registers, and checked findings with healthcare workers; at community level, quarterly review meetings with the senior health surveillance assistants to identify deaths not yet reported; at the end of the month, the lists from the two data sources were compared and duplicates were removed; experienced research staff visited households of all maternal deaths and interviewed all persons (traditional birth attendants, neighbors, relatives with knowledge); verbal autopsy was delayed one month to allow for a period of mourning; if maternal death was at a facility, information pulled from case notes, notes made at time of review of death by facility audit team and interviews with staff who cared for the patient; interviews conducted by two staff members; panel of experts (two independent obstetrician-gynecologists and a midwife) independently assigned cause of death; considered satisfactory if two agreed, if different assignment by each person, there was a panel review meeting and all three needed to agree; a fourth expert was included if needed^15^ - Bi-annual surveillance in which all households were visited with sample of all WRA that resided in the demographic surveillance area from 2000-2009; verbal autopsy conducted on average 9 months are the survey; interviews were conducted by trained nurses; two clinicians independently assigned the underlying cause of death^16^ - Selected deaths from the demographic surveillance system; removed deaths already recently reclassified; families of remaining individuals were visited by a female interviewed; two physicians assigned cause of death; all possible maternal deaths were submitted to a detailed survey wherein relatives were visited by the trained female interviewer; independently reviewed by two physicians who assigned cause of death; disagreements discussed and agreed upon cause of death assigned, if there was no agreement, a third physician joined in for 2/3 agreement^17^ - Data came from the Matlab demographic surveillance system area (conducted in November-December 1991); records of 510 deaths were used; prepared list of surviving brothers and sisters 15+ years old, born to the same mother; siblings (one for each decedent) were interviewed by five specially trained female interviewers; another interview by a trained female investigator for suspected maternal deaths and talked to community health workers who had visited the decedent regularly and knew them well or the decedent's female relatives and neighbors; death records reviewed by an experienced public health physician; cause of death was assigned by the health assistant without looking at the demographic surveillance site cause of death and consulted with the public health physician who supervises demographic surveillance site assignment where necessary ^18,19^ - 15 field assistants trained in mortality inquiry in the community contacted village headman, traditional birth attendants, health workers and volunteers, village watchman (who register births and deaths) and several women in each street of the village and enquired about deaths which occurred in WRA (15-44 years) during the preceding year; surviving family members were interviewed by doctors who completed the questionnaires and identified maternal deaths; a team of doctors visited each family in which maternal death occurred and surviving family members were interviewed; prescription slips and other record of illness in the house were examined, traditional birth attendants and doctors who attended the case were independently interviewed; cause of death assigned by interviewers and hospital records of those who died in hospitals were also reviewed; maternal deaths were discussed by a panel of doctors from Community Medicine and Obstetrics^20^ - Identification of all deaths in the population; census of 16,247 families living in refugee settlements from May - September 2000; community health workers visited each family and recorded the number of living individuals in each household and deaths that occurred in a certain time period; and then an investigation of deaths among WRA identified via lists of refugees who died in each camp and investigated with verbal autopsy questionnaire ; verbal autopsies done by one of two investigators trained in clinical obstetrics along with a translator; in 92% of cases, women who lived with the decedent were interviewed one primary respondent and other auxiliary/complimentary; medical records and medicines also examined^21^ - Compared names from community population registers and hospital registers to list all deaths to WRA in 1994-1995, removed duplicates and investigated each death to determine cause by sending investigators to each province, conducted exhaustive search for additional deaths by meeting with officials of the Ministry of Health and the National Committee for Population and Family Planning at all levels and with commune and village officials of the Woman's Union, investigated each death to determine its cause and whether it was a maternal death using verbal autopsy with relatives listed by provinces and identified by further research; in some cases, interviewed health workers who attended the death; used one questionnaire to determine cause of death and if the deceased individual was pregnant at their death; the next questionnaire for individuals who were pregnant was administered to the principal health care provider to verify information and learn more about medical circumstances around the death^22^ - Identified deaths through vital registers and health centers, where death certificates are issues; research staff visited health care facilities and vital registers; compared and found identical; family of the decedent was contacted and research team of four trained female social workers not employed by health services interviewed after three months (period of mourning) using a verbal autopsy; structured questionnaires with one to five family members, colleagues, friends, and traditional healers; repeated until steps leading to death clarified; post-mortems conducted if a crime was suspected; group of senior physicians and authors analyzed all data to assign the cause of death^5^ | **3** |
| *Definitions* | Uses ICD-9 definitions, plus some modifications for obstetrician-gynecologist causes | - "Occurred during pregnancy... or within 3 months following pregnancy termination"^23^ - ICD-9 but postpartum through 40 days - "Any deaths to women aged 15-50" but used ICD-9 coding and eight general-cause categories^2^ - Women aged 15-50 years coded as a maternal death or occurring within 90 days of the date of birth of the child^17^ - WRA (15-44 years)^20^ - Maternal and late maternal deaths, i.e., within a year^21^ | **4** |
| *Sample size* | Does not report sample size | - 1,037 deaths^23^ - 10.135 households^6,7^ - 38,563 households^8^ - 46 deaths^9^ - 156 deaths^10^ - 1,001 verbal autopsies^11–13^ - 997 deaths, 841 interviewed^2^ - 198,989 individuals in catchment area and 965 deaths^14^ - 424 deaths^15^ - 15,526 households^16^ - 795 deaths, 204 potential maternal deaths, 196 ultimately classified as maternal death^17^ - 510 deaths and 401 who had family members listed, 384 respondents, with 305 reporting a deceased sibling^18,19^ - 257 deaths^20^ - 16,247 families and 1,197 deaths^21^ - 2,886 deaths, 529 removed, 465 additional found, 321 identified as maternal^22^ - 97 deaths^5^ | **1** |
| *Timing of point estimate relative to data collection* | 2-3 years of prospective implementation; produces age specific mortality rates for 100,000 married women | - Reviewed deaths from past 10 years^23^ - Reviewed deaths from past 5 years^6,7^ - Review of 3.5 years (January 2005-July 2009) of maternal deaths^9^ - Reviewed deaths from the past 12 months^10^ - Reviewed deaths from past 3 years^11–13^ - Reviewed deaths from past 1 year^20^ - Reviewed deaths from past 6 years^17^ - Reviewed deaths from past 15 years^18,19^ - Reviewed deaths from January 20, 1999 - August 31, 2000^21^ - Reviewed deaths from past 2 years^22^ - Reviewed deaths during 1 year^2,14,15^ - Reviewed deaths during 2 years^5^ | **1** |
| *Bias* | - Selection bias (only asks about ever-married women of reproductive age) - Selection bias: estimate that they only found 45-50% of deaths (and had to adjust) - Validation: retested with a 10% random sample [good] - Found that it was reliable compared to other methods - Report that locations were selected because there was a prevalence of contraception, expected number of deaths sufficient for analysis, and likelihood of locating a high proportion of deaths | - Used several different approaches to measuring/estimating the number of lives births in a year^15^ - Local event calendars used to help with recall^20^ | **2** |
|  |  |  |  |
| *Human resources* | Does not report on human resources required | - CHW interviews, physician reviews^23^ - Verbal autopsy by female doctors, three independent obstetricians to assign cause of death^6,7^ - Lady health visitor or female medical technician surveys (2) and interviews (n = ?), review by senior obstetricians and family physicians^8^ - Two house listers, two research investigators to validate lists, one doctor assigned cause of death, then external reviewer for cause of death (reviewer was gynecologist or two physicians and a surgeon)^10^ - 10 local fieldworkers, clinician assessment, two physicians to assign cause of death, sometimes a third^11–13^ - Clerks, social workers^2^ - Notification assistants, research assistant, two physicians^14^ - Meeting leads, providers, research staff, senior surveillance assistants, two staff members per interview, two obstetrician-gynecologists and a midwife to review cause of death, sometimes with a fourth expert^15^ - Trained nurses, two clinicians^16^ - Female interviewers; two physicians for cause of death, sometimes a third expert^17^ - Five specially trained interviewers, community health workers, public health physician, public health assistant^18,19^ - 15 field assistants, doctor interviewers, panel of doctors to assign cause of death^20^ - Community health workers, two investigators and a translator^21^ - Research staff, four trained social workers group of senior physicians and authors^5^ | **1** |
| *Time needed for implementation* | 3 years in Egypt and 2 years in Indonesia | - First round of interviews, 4-12 weeks, then second round (Maternal mortality in different) - 1.5 months of death reviews (July 1 - August 15, 2009) (Improving maternal mortality) - List preparation tool 3 months (June - August 2003)^10^ | **2** |
| *Data collection training* | Survey questionnaire for all deaths (for use with husband, mother, or mother-in-law) | NA | **2** |
| *Statistical training* | Does not provide calculation, especially given adjustments based on other methods | NA | **3.5** |
| *Digitalization* | Easy to digitize | NA | **4** |
| *Cost* | Cost not reported | NA | **2** |
| *Total score* | | | **23.5/44** |

**References**

1. Fortney JA, Gadalla S, Saleh S, Susanti I, Potts M, Rogers SM. Causes of death to women of reproductive age in two developing countries. *Popul Res Policy Rev*. 1987;6(2):137-148. doi:10.1007/BF00149205

2. Fortney JA, Susanti I, Gadalla S, Saleh S, Feldblum PJ, Potts M. Maternal mortality in Indonesia and Egypt. *International Journal of Gynecology & Obstetrics*. 1988;26(1):21-32.

3. Grubb GS, Fortney JA, Saleh S, et al. A comparison of two cause-of-death classification systems for deaths among women of reproductive age in Menoufia, Egypt. *Int J Epidemiol*. 1988;17(2):385-391. doi:10.1093/ije/17.2.385

4. Fortney JA, Susanti I, Gadalla S, Saleh S, Rogers SM, Potts M. Reproductive mortality in two developing countries. *Am J Public Health*. 1986;76(2):134-138. doi:10.2105/AJPH.76.2.134

5. Wessel H, Reitmaier P, Dupret A, Rocha E, Cnattingius S, Bergström S. Deaths among women of reproductive age in Cape Verde, causes and avoidability. *Acta Obstetricia et Gynecologica Scandinavica*. 1999;78(3):225-232.

6. Fikree FF, Karim MS, Midhet F, Berendes HW. Causes of reproductive age mortality in low socioeconomic settlements of Karachi. *Journal of Pakistan Medical Association*. 1993;43(10):208.

7. Fikree FF, Gray RH, Berendes HW, Karim MS. A community-based nested case-control study of maternal mortality. *International Journal of Gynecology & Obstetrics*. 1994;47(3):247-255.

8. Fikree F, Midhet F, Sadruddin S, Berendes H. Maternal mortality in different Pakistani sites: Ratios, clinical causes and determinants. *Acta obstetricia et gynecologica Scandinavica*. 1997;76:637-645. doi:10.3109/00016349709024603

9. Geynisman J, Latimer A, Ofosu A, Anderson FWJ. Improving maternal mortality reporting at the community level with a 4-question modified reproductive age mortality survey (RAMOS). *Int J Gynaecol Obstet*. 2011;114(1):29-32. doi:10.1016/j.ijgo.2011.01.011

10. Iyengar K, Iyengar SD, Suhalka V, Dashora K. Pregnancy-related deaths in rural Rajasthan, India: exploring causes, context, and care-seeking through verbal autopsy. *Journal of health, population, and nutrition*. 2009;27(2):293.

11. Kahn K, Tollman SM, Garenne M, Gear JS. Validation and application of verbal autopsies in a rural area of South Africa. *Tropical Medicine & International Health*. 2000;5(11):824-831.

12. Kahn K, Tollman S, Garenne M, Gear J. Validation and application of verbal autopsies in a rural area of South Africa | Request PDF. *Tropical Medicine & International Health*. Published online 2000. Accessed April 7, 2023. https://www.researchgate.net/publication/12204685_Validation_and_application_of_verbal_autopsies_in_a_rural_area_of_South_Africa

13. Kahn K, Tollman SM, Garenne M, Gear JS. Who dies from what? Determining cause of death in South Africa’s rural north-east. *Tropical Medicine & International Health*. 1999;4(6):433-441.

14. Khoury SA, Massad D, Fardous T. Mortality and causes of death in Jordan 1995-96: assessment by verbal autopsy. *Bulletin of the World Health Organization*. 1999;77(8):641.

15. Mgawadere F, Unkels R, Adegoke A, van den Broek N. Measuring maternal mortality using a Reproductive Age Mortality Study (RAMOS). *BMC Pregnancy and Childbirth*. 2016;16(1):291. doi:10.1186/s12884-016-1084-8

16. Nabukalu D, Klipstein-Grobusch K, Herbst K, Newell ML. Mortality in women of reproductive age in rural South Africa. *Global Health Action*. 2013;6(1):22834.

17. Ronsmans C, Vanneste A, Chakraborty J, Ginneken J. A comparison of three verbal autopsy methods to ascertain levels and causes of maternal deaths in Matlab, Bangladesh. *International journal of epidemiology*. 1998;27:660-666. doi:10.1093/ije/27.4.660

18. Shahidullah M. A comparison of sisterhood information on causes of maternal death with the registration causes of maternal death in Matlab, Bangladesh. *Int J Epidemiol*. 1995;24(5):937-942. doi:10.1093/ije/24.5.937

19. Shahidullah M. The sisterhood method of estimating maternal mortality: the Matlab experience. *Stud Fam Plann*. 1995;26(2):101-106.

20. Kumar R, Sharma AK, Barik S, Kumar V. Maternal mortality inquiry in a rural community of north India. *International Journal of Gynecology & Obstetrics*. 1989;29(4):313-319.

21. Bartlett LA, Jamieson DJ, Kahn T, Sultana M, Wilson HG, Duerr A. Maternal mortality among Afghan refugees in Pakistan, 1999-2000. *The Lancet*. 2002;359(9307):643-649.

22. Hanenberg R, Vach T, Vinh D, Sokal D. Maternal mortality in Vietnam in 1994-95. *Studies in family planning*. 2000;30:329-338.

23. Fauveau V, Chakraborty J. Maternity care in Matlab: Present status and possible interventions. Published online 1988.
