# Supplementary material for: Maternal mortality estimation methodologies: a scoping review and evaluation of suitability for use in humanitarian settings
Source: Confl Health. 2024 Dec 19;18:75. doi: 10.1186/s13031-024-00636-y (PMC11657123; doi:10.1186/s13031-024-00636-y)
Supplement: Supplementary file 9 — Additional file 9. Census- or survey-based methodology completed evaluation form. Additional file 9 shows the completed evaluation form for the census- or survey-based methodology. [file 13031_2024_636_MOESM9_ESM.docx]

**Additional file 9. Census- or survey-based methodology completed evaluation form**

| **Category** | **Census-/survey-based method** | | |
| --- | --- | --- | --- |
|  | **Notes from original implementation** | **Notes from additional implementations** | **Score (1-4)** |
| *Summary of methodology* | Mortality data are measured through regularly implemented and exhaustive, nationally-representative surveys or censuses; these censuses and surveys rely on accurate vital statistics and civil registration data | | |
| *Data sources* | NA | - Nationally-representative household surveys in three countries; specifically looking for age and sex distributions from two censuses separated by no more than 15 years, household deaths by age and sex for a defined time period before one or both of the censuses, for deaths of women of reproductive age, whether the woman was pregnant or within 6 weeks of the end of the pregnancy when she died, information on births by age of the mother in a defined time period before one or both censuses, and the number of children ever born to each woman^1^ - Bangladesh Maternal Health Services and Maternal Mortality Survey^2^ - Requires a census to report on the number of deaths of WRA in a defined period, the proportion of deaths that are pregnancy-related, and the number of births in the same time period^3^ - Requires extensive cartographic mapping exercises to subdivide countries into standard enumeration areas; two-stage survey implementation^4^ | **2** |
| *Definitions* | NA | - Pregnancy-related mortality rate (i.e., includes non-maternal deaths; any death during pregnancy or within 42 days)^1^ - Deaths to women 13-49 years old and within 2 months of birth^2^ - Deaths to women 15-49 years old and within 2 months or 42 days of birth^3^ | **3** |
| *Sample size* | NA | - 99,202 households interviewed, including 103,796 ever-married women^2^ - Aimed to have a geographically representative sample of 900,000 individuals^5^ | **1** |
| *Timing of point estimate relative to data collection* | NA | - Estimate covers 7 years or more^1^ - Asked about deaths in the past few years (implemented in 2001 and asked about deaths after April 1997)^2^ - Some methodologies estimate deaths in the intercensal period^1,3,4^ | **2** |
| *Bias* | NA | - Assumptions in the model:   - Data are accurate/reliable   - Closed population (no migration)   - Completeness of recording of deaths is constant across ages   - Completeness of recording of population is constant across ages   - Ages of the living and dead are reported without error^1,3,4^ - Large sample sizes are needed^6^ - Ever-married women only^2^ | **2** |
|  |  |  |  |
| *Human resources* | NA | - 4,800 to 22,225 enumerators across five censuses^6^ - Required coordination from the Bangladesh National Institute for Population Research and Training, Johns Hopkins University, International Centre for Diarrhoeal Disease Research, Bangladesh, and ORC Macro^2^ - 25,000 enumerators^4^ - 337 health extension workers and 34 primary healthcare unit supervisors; first stage required 2,500 person-days of work; 132 health extension workers for the second step and 100 person-days of work^5^ | **1** |
| *Time needed for implementation* | NA | - One to three days of training, plus 2,600 person-days of work^5^ | **1** |
| *Data collection training* | NA | - One week or less to 2-4 weeks across five census implementations^6^ - One-day training program conducted by two study coordinators; two days of training for the second step^5^ | **2** |
| *Statistical training* | NA | - Use General Growth Balance method from the demographic balancing equation to understand how well reported deaths cover the population^1^ - Calculated age-specific all-cause mortality through comparisons with the Matlab demographic surveillance site^2^ - Use General Growth Balance and Synthetic Extinct Generations to calculate deaths in the intercensal period^3,4^ | **3.5** |
| *Digitalization* | NA | - Census questions could be digitalized, as could the calculation of maternal mortality^1–6^ | **4** |
| *Cost* | NA | - US$60,000.00^5^ - Expected to be high given large sample sizes and human resources needed^1–6^ | **1** |
| *Total score* | | | **22.5/44** |

**References**

1. Hill K, Queiroz BL, Wong L, et al. Estimating pregnancy-related mortality from census data: experience in Latin America. *Bull World Health Organ*. 2009;87(4):288-295. doi:10.2471/blt.08.052233

2. Hill K, El Arifeen S, Koenig M, Al-Sabir A, Jamil K, Raggers H. How should we measure maternal mortality in the developing world? A comparison of household deaths and sibling history approaches. *Bull World Health Organ*. 2006;84(3):173-180. doi:10.2471/blt.05.027714

3. Hill K, Stanton C. Measuring maternal mortality through the census: rapier or bludgeon? *Journal of Population Research*. 2011;28:31-47.

4. Banda R, Fylkesnes K, Sandøy IF. Rural-urban differentials in pregnancy-related mortality in Zambia: estimates using data collected in a census. *Popul Health Metr*. 2015;13:32. doi:10.1186/s12963-015-0066-9

5. Godefay H, Abrha A, Kinsman J, Myléus A, Byass P. Undertaking cause-specific mortality measurement in an unregistered population: an example from Tigray Region, Ethiopia. *Glob Health Action*. 2014;7:25264. doi:10.3402/gha.v7.25264

6. Stanton C, Hobcraft J, Hill K, et al. Every death counts: measurement of maternal mortality via a census. *Bulletin of the World Health Organization*. 2001;79:657-664.
