# Supplementary material for: Maternal mortality estimation methodologies: a scoping review and evaluation of suitability for use in humanitarian settings
Source: Confl Health. 2024 Dec 19;18:75. doi: 10.1186/s13031-024-00636-y (PMC11657123; doi:10.1186/s13031-024-00636-y)
Supplement: Supplementary file 10 — Additional file 10. Direct sisterhood methodology completed evaluation form. Additional file 10 shows the completed evaluation form for the direct sisterhood methodology. [file 13031_2024_636_MOESM10_ESM.docx]

**Additional file 10. Direct sisterhood methodology completed evaluation form**

| **Category** | **The direct sisterhood method (Rutenberg, et al, 1991)**^1^ | | |
| --- | --- | --- | --- |
|  | **Notes from original implementation** | **Notes from additional implementations** | **Score (1-4)** |
| *Summary of methodology* | List all brothers and sisters of the respondent and then obtain information on the survivorship of each, ages of the surviving siblings, the ages and years ago of death of decedents, and for each deceased sister, if the death was due to maternal causes | | |
| *Data sources* | DHS data add-on of three questions | - Household Questionnaire with a section on deaths of usual residents of the household; if a death was reported, additional information; three questions about the decreased person if 15-49 years old at time of birth^2^ - Household questionnaire about all household deaths, follow up for women 13-49 years old^3^ - Population-based survey with questions on deaths with households with at least one WRA or one child born there in the last three years; one woman selected from each household based on who had most recently celebrated their birthday^4^ - Cross-sectional, community-based household survey; randomly selected one male or female from each household (if no one, they returned, and if still no one, went to next eligible household and so on until a respondent was found)^5^ - Used data from the 2013 Nigeria DHS^6^ - Nepal 2003 World Health Survey^7^ - 2008 and 2013 Nigeria DHS and data from women who had been pregnant and had given birth to at least one child in Nigeria^8^ - 2004 and 2011 Cameroon DHS and data from women who had been pregnant and had given birth to at least one child in Cameroon^9^ - 2013 Namibia DHS, 2014 Kenya DHS, 2013 Sierra Leone DHS^10^ - Cross-sectional study wherein if more than one person in the household was born to the same mother, one was selected by lottery for inclusion^11^ - 2010 Bangladesh Maternal Mortality Survey^2^ | **2** |
| *Definitions* | Maternal death is within two months after birth or termination; any death in the time period was considered a death, whether related to accident, injury, or maternal cause | - Sorted pregnancy and non-pregnancy related deaths in the Bolivia data^12^ - Death while pregnant or within 42 days of delivery or termination^13^ - Only ever-married sisters^5^ - Death while pregnant or within 60 days of delivery and 13-49 years^3^ - ICD-10^4^ - Death for siblings 12+ years old and during pregnancy, delivery, or two months following delivery^6^ - Deaths during pregnancy or within 42 days for any sister that reached at least 15 years of age^11,14^ - Pregnancy-related (during pregnancy or within 42 days of birth or termination) and maternal death (during pregnancy or within 42 days of termination of pregnancy and related to pregnancy) for individuals 13-49 years old^2^ | **3** |
| *Sample size* | - In Bolivia, 7,923 interviews of WRA - In Sudan, 5,860 interviews of ever-married WRA | - 104,434 households selected for participation and 99,202 participated^3^ - Planned sample size of 2,400 households and had 1,985 participants^4^ - 3,365 women and 598 men interviewed^5^ - 38,948 interviews and 3,302 deaths^6^ - 3,933 WRA respondents who reported 15,344 sisters and 144 maternal deaths^7^ - 820 participants answered four required questions^14^ - 26,082 WRA participants^9^ - 8,880 households and 17,444 respondents; 2,402 deaths and 776 pregnancy-related deaths^11^ - 175,000 households^2^ | **1** |
| *Timing of point estimate relative to data collection* | In Bolivia, 14 years prior to the survey | - Three-year interval estimates up to the year of the survey^13^ - Recall period of one year for deaths^5^ - Seven years of recall^10^ - Five years of recall^11^ - Deaths since October 2006 (4 years, 10 months before survey began)^2^ | **2** |
| *Bias* | - No assumptions needed to convert collected data into estimates of maternal mortality - Deaths listed in chronological order to elicit more complete recording - Probed when respondents said they didn't know the age or year of death of decedents, because bad data is better than missing data - Decedent must have surviving sibling, that was with the person when they died, and knows that it was a maternal death, and knows the cause of death - Underreporting of earlier deaths due to recall bias - Selection bias of who is available to respond to a survey | - Interviews with only female respondents omits decedents that do not have female siblings^3,4,6,8,9,12–14^ - If only interviewing one sibling and many are available, data cannot be validated through cross-checking^4,5^ | **2** |
|  |  |  |  |
| *Human resources* | Not reported | - Three local female field assistants conducted household surveys^14^ | **1** |
| *Time needed for implementation* | 8-10 minutes per interview  Conducted in Bolivia between March and July 1989 | - Six months of data collection^13^ - May 2013 - June 2013: 2 months^4^ - Data collection from February to March 2012^5^ - Seven months of data collection in a population of 23,600^14^ - July 2019 - May 2020: 11 months^11^ - January 2010 - August 2010: 8 months^2^ | **1** |
| *Data collection training* | Four additional questions in the DHS questionnaire; not additional training reported | NA | **2** |
| *Statistical training* | Calculate maternal mortality by computing the number of person-years of exposure to maternal mortality (i.e., the total number of years all sisters lived during some time period) and the number of maternal deaths by time period; maternal mortality rates calculated by dividing the number of deaths by the person-years of exposure; maternal mortality rate divided by the general fertility rate fives the maternal mortality ratio; since time and age at death for each sister is known, estimates can be made for a series of time periods and for women by age-group | Complicated calculations to disentangle pregnancy and non-pregnancy related deaths^12^ | **3.5** |
| *Digitalization* | Easy to digitize | Collected on ODK^4^ | **4** |
| *Cost* | Not reported | NA | **1** |
| *Total score* | | | **22.5/44** |

**References**

1. Rutenberg N, Sullivan JM. Direct and Indirect Estimates of Maternal Mortality. In: *Demographic and Health Surveys World Conference*. Vol 3. IRD/Macro International, Incorporated; 1991:1669.

2. Singh K, Li Q, Ahsan KZ, Curtis S, Weiss W. A comparison of approaches to measuring maternal mortality in Bangladesh, Mozambique, and Bolivia. *Popul Health Metr*. 2022;20(1):5. doi:10.1186/s12963-022-00281-8

3. Hill K, El Arifeen S, Koenig M, Al-Sabir A, Jamil K, Raggers H. How should we measure maternal mortality in the developing world? A comparison of household deaths and sibling history approaches. *Bull World Health Organ*. 2006;84(3):173-180. doi:10.2471/blt.05.027714

4. Moseson H, Massaquoi M, Bawo L, et al. Estimation of maternal and neonatal mortality at the subnational level in Liberia. *Int J Gynaecol Obstet*. 2014;127(2):194-200. doi:10.1016/j.ijgo.2014.05.007

5. Anastasi E, Ekanem E, Hill O, Adebayo Oluwakemi A, Abayomi O, Bernasconi A. Unmasking inequalities: Sub-national maternal and child mortality data from two urban slums in Lagos, Nigeria tells the story. *PLoS One*. 2017;12(5):e0177190. doi:10.1371/journal.pone.0177190

6. Ariyo O, Ozodiegwu ID, Doctor HV. The influence of the social and cultural environment on maternal mortality in Nigeria: Evidence from the 2013 demographic and health survey. *PLoS One*. 2017;12(12):e0190285. doi:10.1371/journal.pone.0190285

7. Bhandary S. Evaluating maternal mortality ratios of Nepal with 2003 Nepal world health survey estimates. *Journal of General Practice and Emergency Medicine of Nepal*. 2018;5(7):15-19.

8. Meh C, Thind A, Ryan B, Terry A. Levels and determinants of maternal mortality in northern and southern Nigeria. *BMC Pregnancy Childbirth*. 2019;19(1):417. doi:10.1186/s12884-019-2471-8

9. Meh C, Thind A, Terry AL. Ratios and determinants of maternal mortality: a comparison of geographic differences in the northern and southern regions of Cameroon. *BMC Pregnancy Childbirth*. 2020;20(1):194. doi:10.1186/s12884-020-02879-y

10. Izugbara C. Age differentials in pregnancy-related deaths in selected African countries. *J Obstet Gynaecol*. 2021;41(4):516-521. doi:10.1080/01443615.2020.1754367

11. Kea A, Lindtjorn B, Tekele A, Hinderaker S. Reduction in maternal mortality ratio varies by district in Sidama Regional State, southern Ethiopia: Estimates by cross-sectional studies using the sisterhood method and a household survey of pregnancy and birth outcomes. Published online October 4, 2022. doi:10.1101/2022.10.02.22280613

12. Stecklov G. Maternal mortality estimation: separating pregnancy-related and non-pregnancy-related risks. *Stud Fam Plann*. 1995;26(1):33-38.

13. Hill KL, El-Arifeen S, Chowdhury HR, Rahman S. Adult female mortality: levels and causes. *Bangladesh maternal health services and maternal mortality survey 2001*. Published online 2001.

14. Liese KL, Pauls H, Robinson S, Patil C. Estimating Maternal Mortality in Remote Rural Regions: an Application of the Sisterhood Method in Tajikistan. *Cent Asian J Glob Health*. 2019;8(1):341. doi:10.5195/cajgh.2019.341
